# Genetic Analysis of Six Transmembrane Protein Family Genes in Parkinson’s Disease in a Large Chinese Cohort

**DOI:** 10.3389/fnagi.2022.889057

**Published:** 2022-07-04

**Authors:** Yuwen Zhao, Kailin Zhang, Hongxu Pan, Yige Wang, Xiaoxia Zhou, Yaqin Xiang, Qian Xu, Qiying Sun, Jieqiong Tan, Xinxiang Yan, Jinchen Li, Jifeng Guo, Beisha Tang, Zhenhua Liu

**Affiliations:** ^1^Department of Neurology, Xiangya Hospital, Central South University, Changsha, China; ^2^National Clinical Research Center for Geriatric Disorders, Xiangya Hospital, Central South University, Changsha, China; ^3^Department of Geriatrics, Xiangya Hospital, Central South University, Changsha, China; ^4^Centre for Medical Genetics and Hunan Key Laboratory of Medical Genetics, School of Life Sciences, Central South University, Changsha, China; ^5^Key Laboratory of Hunan Province in Neurodegenerative Disorders, Central South University, Changsha, China

**Keywords:** TMEM protein family genes, genetic analysis, Parkinson’s disease, burden analysis, rare variants, common variants

## Abstract

**Objectives:**

Parkinson’s disease (PD) is a neurodegenerative disorder with the manifestation of motor symptoms and non-motor symptoms. Previous studies have indicated the role of several transmembrane (TMEM) protein family genes in PD pathogenesis.

**Materials and Methods:**

In order to better investigate the genetic role of PD-related TMEM protein family genes in PD, including *TMEM230*, *TMEM59*, *TMEM108*, *TMEM163*, *TMEM175*, and *TMEM229B*, 1,917 sporadic early onset PD (sEOPD) or familial PD (FPD) patients and 1,652 healthy controls were analyzed by whole-exome sequencing (WES) while 1,962 sporadic late-onset PD (sLOPD) and 1,279 healthy controls were analyzed by whole-genome sequencing (WGS). Rare and common variants for each gene were included in the analysis.

**Results:**

One hundred rare damaging or loss of function variants of six genes were found at the threshold of MAF < 0.1%. Three rare Dmis variants of *TMEM230* were specifically identified in PD. Rare missense variants of *TMEM59* were statistically significantly associated with PD in the WES cohort, indicating the role of *TMEM59* in FPD and sEOPD. Rare missense variants of *TMEM108* were suggestively associated with PD in the WGS cohort, indicating the potential role of *TMEM108* in sLOPD. The rare variant of the other three genes and common variants of six genes were not significantly associated with PD.

**Conclusion:**

We performed a large case-control study to systematically investigate the role of several PD-related TMEM protein family genes in PD. We identified three PD-specific variants in *TMEM230*, the significant association of *TMEM59* with FPD, and sEOPD and the suggestive association of *TMEM108* with sLOPD.

## Introduction

Parkinson’s disease (PD) is the second most prevalent neurodegenerative disorder characterized by different combinations of motor and non-motor symptoms, including bradykinesia, resting tremor, rigidity, postural instability, olfactory loss, sleep dysfunction, autonomic dysfunction, and cognitive impairment (Armstrong and Okun, 2020). The pathophysiology of PD is dopaminergic neuron death and α-synuclein accumulation (Bloem et al., 2021). Neuroinflammation, mitochondrial/oxidative stress pathways dysregulation, autophagy/lysosomal dysfunction, and synaptic vesicle endocytosis disruptions have been indicated in the pathogenesis of PD (Nguyen et al., 2019; Hirsch and Standaert, 2021). Genetic, epigenetic, environmental, and lifestyle risk factors contribute to PD etiology (Jankovic and Tan, 2020). Previous studies have identified more than 20 PD-causing genes through linkage analysis or homozygous mapping strategies and many PD risk loci affecting disease risk, age at onset (AAO), and progression of PD through the genome-wide association study (GWAS) (Blauwendraat et al., 2020).

Transmembrane (TMEM) proteins belong to a large protein family with the characteristic of having at least one transmembrane peptide that entirely or partially crosses the biological membrane (Marx et al., 2020). With the transmembrane property, TMEM proteins play an essential role in many cell processes, such as signal transduction, ion/small molecule transportation, and cell adhesion (Ryu et al., 2019). Especially some members of the TMEM protein family play roles in PD-related pathways, such as synaptic vesicle trafficking and lysosomal and mitochondrial function (Deng et al., 2016; Jinn et al., 2017; Wang et al., 2021a). However, many TMEM proteins are still poorly investigated (Marx et al., 2020).

Several TMEM proteins have been reported to be associated with PD, for example, *TMEM230*, *TMEM175*, *TMEM163*, *TMEM229B*, *TMEM108*, and *TMEM59*. *TMEM230* mutations were reported to cause autosomal dominant PD (Deng et al., 2016). Moreover, mutant TMEM230 could impair synaptic vesicle trafficking, disrupt mitochondria transport, and induce apoptotic cell death (Deng et al., 2016; Wang et al., 2020, 2021a). A genome-wide significant locus (rs34311866) located around the *TMEM175* gene was reported in a large-scale GWAS meta-analysis study (Nalls et al., 2014), and *TMEM175* deficiency resulted in lysosomal and mitochondrial dysfunction and α-synuclein aggregation (Jinn et al., 2017). Another two loci located around *TMEM163* or *TMEM229B* were also identified to be associated with PD in a large-scale meta-analysis of the GWAS study (Nalls et al., 2014). In contrast, a large longitudinal study found the rs138073281 located around *TMEM108* linked to cognitive progression in PD (Zhang et al., 2021). Specifically, the association of *TMEM163* with PD was further confirmed in other cohorts (Kia et al., 2021; Rudakou et al., 2021). *TMEM59* was reported within the PARK10 locus indicating a possible role in PD (Beecham et al., 2015). Moreover, over-expression of *TMEM59* in a *Drosophila* PD model ameliorated the phenotype of shortened lifespan, impaired locomotor activity, and dopaminergic neuron loss (Zhang et al., 2018). Although the above evidence indicated that those TMEM proteins family genes might be associated with PD, few studies systematically analyze whether these genes are associated with early onset or late-onset PD populations, respectively, considering that early onset and late-onset cases may have certain genetic heterogeneity.

This study analyzed rare and common variants of *TMEM230*, *TMEM175*, *TMEM163*, *TMEM229B*, *TMEM108*, and *TMEM59* with gene-based and allele-based, respectively, through next-generation sequencing in a large Chinese PD cohort to better explore the genetic contribution of these six TMEM protein family genes in Chinese PD patients.

## Materials and Methods

### Participants

Three thousand eight hundred seventy-nine unrelated PD patients and 2,931 healthy controls were enrolled in this study. Blood samples for DNA extraction and clinical data of each participant were collected. All the participants have been divided into two cohorts: 1,917 sporadic early onset PD (sEOPD) or familial PD (FPD) probands and 1,652 healthy controls were in one cohort, and 1,962 sLOPD probands and 1,279 healthy controls were in another cohort (Supplementary Table 1). PD diagnosis was made according to the Movement Disorders Society (MDS) clinical diagnostic criteria by neurologists with expertise in movement disorders at the Department of Neurology, Xiangya Hospital, and other centers from Parkinson’s Disease & Movement Disorders Multicenter Database and Collaborative Network in China (PD-MDCNC).^1^ This study conforms with the World Medical Association Declaration of Helsinki. Each participant signed the written consent. The Ethics Committee of Xiangya Hospital, Central South University, approved the study. It is worth noting that the PD patients harboring the pathogenic or likely pathogenic variants identified in sEOPD or FPD patients were ruled out in this study, according to the principle of our previous study (Zhao et al., 2020).

### Gene Selection

We included six TMEM protein family genes to perform the genetic analysis in this study (Supplementary Table 2). *TMEM230* was reported to cause autosomal dominant PD; *TMEM175*, *TMEM163*, and *TMEM229B* were found to be risk loci of PD in a large meta-analysis GWAS study; *TMEM108* was associated with cognitive progression in PD; *TMEM59* was functionally related to PD phenotype. To investigate the role of these six TMEM protein family genes in PD, we conducted the genetic analysis by next-generation sequencing in FPD, sEOPD, sLOPD patients, and healthy controls from a large Chinese cohort.

### Sequencing and Bioinformatic Analysis

We sequenced the first cohort with 1,917 sEOPD or FPD patients and 1,652 healthy controls by whole-exome sequencing (WES) with an average sequencing depth of 123-fold using the HiSeq X10 platform; the second cohort with 1,962 sLOPD and 1,279 healthy controls were sequenced by whole-genome sequencing (WGS) with an average sequencing depth of 12-fold using the HiSeq Nova platform. Variants calling for those six TMEM protein family genes were done by the following methods or algorithms: Burrows-Wheeler Aligner-MEM algorithm (Li, 2014), Picard,^2^ and Genome Analysis Toolkit (Van der Auwera et al., 2013). Annotation for the variants was accomplished by ANNOVAR and VarCards. The pathogenicity of missense variants was predicted by the Combined Annotation Dependent Depletion (CADD) algorithm, similar to a previous publication. Variants from *TMEM230*, *TMEM175*, *TMEM163*, *TMEM229B*, *TMEM108*, and *TMEM59* were then filtered by the same strategy used in our previous study (Pan et al., 2020). After the variants filtering process, rare missense variants, rare damaging missense (Dmis) variants, and loss-of-function (LoF) variants were used for the gene-based burden analysis. The single-variant logistic association analysis included all common variants in the coding regions and upstream/downstream 2000 bp of each gene. We conducted Sanger sequencing on several random rare damaging variants, and the results showed that these variants are actual (Supplementary Figure 1). Moreover, we performed Sanger sequencing for the available family member of corresponding families with rare damaging variants predicted by two algorithms (CADD score over 12.37 and ReVe score over 0.7). ReVe is another algorithm used for predicting the deleteriousness of the missense variants (Li et al., 2018).

### Statistical Analysis

We gathered the rare missense variants, rare Dmis variants, or LoF variants, respectively, and performed gene-based burden analysis for each TMEM gene enrolled. We used the sequence kernel association test (SKAT) R package to run the optimized sequence kernel association test (SKAT-O) and calculate the association of different categories of variants from each gene with PD. We included sex and the first five principal components of ancestry as covariates to account for the potential confounding effects in the analysis for the WES cohort, and we also included an extra covariate, age, in the analysis for the WGS cohort. The gene-based burden analysis was done for each gene in each cohort at the threshold of minor allele frequency (MAF) < 1% or MAF < 0.1%. Bonferroni correction was used to adjust the multiple testing, and *P* < 0.0083 was regarded as statistically significant as six independent tests were performed in this study. The result with *P*-value between 0.0083 and 0.05 was considered “suggestively” significant in analyzing of the rare variants.

For the analysis of the common variants, PLINK was used to perform the logistic regression analysis in the WES cohort and WGS cohort, respectively, with the similar covariates mentioned above. Similarly, *P* < 0.0014 was considered statistically significant in the WES cohort (36 independent tests), whereas *P* < 0.000035 in the WGS cohort (1,444 independent tests).

## Results

This study mined the WES or WGS data of 3,879 PD patients and 2,931 healthy controls. There were 1,917 sEOPD or FPD patients and 1,652 healthy controls in the first cohort with WES. The sEOPD or FPD patients were at the mean age of 52.22 ± 9.03 years with a mean AAO of 46.31 ± 8.40 years, and the healthy controls were at the mean age of 62.03 ± 12.59 at the time of enrollment. There were 1,962 sLOPD and 1,279 healthy controls for WGS in the second cohort. The mean age for the sLOPD patients and their healthy matching controls was 66.76 ± 7.08 years and 62.32 ± 7.11 years, respectively. In addition, the mean AAO for sLOPD at the enrollment was 61.88 ± 6.93 years (Supplementary Table 1).

A total of 118 rare missense and eight rare LoF variants were found in the WES cohort, whereas 111 rare missense and seven rare LoF variants were detected in the WGS cohort, at the threshold of MAF < 1%. Furthermore, 102 and 94 rare missense variants, and seven and six rare LoF variants were found in the WES and WGS data, respectively, at the threshold of MAF < 0.1% (Supplementary Table 3). On the other hand, for the WES cohort, there were 36 common variants of these six genes included in the single-variant logistic association analysis. In contrast, 1,444 common variants of these six genes included in the WGS cohort. However, they were not significantly associated with PD in our cohorts.

**FIGURE 1:**
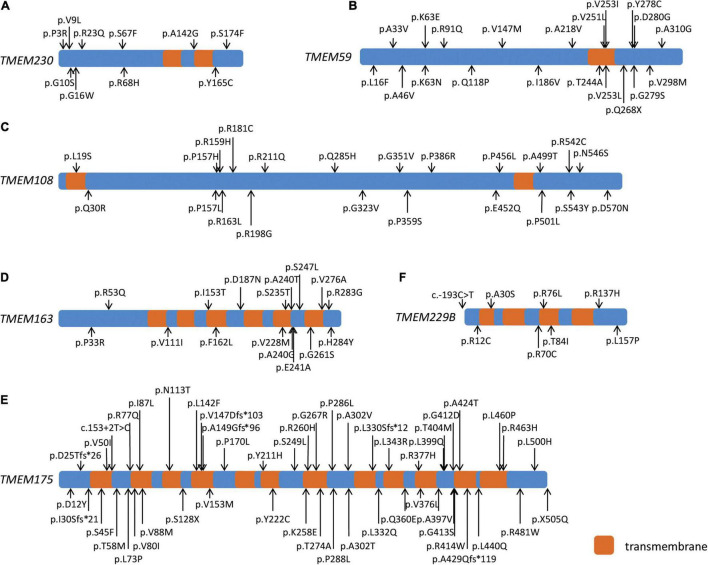
Schematic diagram of six PD-related transmembrane (TMEM) protein family genes. Schematic diagram showing the transmembrane regions of six PD-related TMEM protein family genes and the rare damaging missense and loss-of-function variants of *TMEM230* **(A)**, *TMEM59* **(B)**, *TMEM108* **(C)**, *TMEM163* **(D)**, *TMEM175* **(E)**, and *TMEM229B* **(F)** in both cohorts.

We detected nine missense variants, including six Dmis variants for *TMEM230* with MAF < 1% in the WES, whereas 10 missense variants and six Dmis variants were identified in the WGS cohort. At the threshold of MAF < 0.1%, six or seven missense variants were found in the WES or WGS cohort, with four of them being Dmis variants in each cohort (Table 1; Figure 1A). Gene-based burden analysis showed no significant association of these variants with PD. However, three rare Dmis variants, including p.S174F, p.Y165C, and p.V9L were identified in four sporadic PD patients from the WGS cohort but not in control individuals of our two cohorts (Table 2).

**TABLE 1:** Analysis of rare variant burden within targeted genes in Parkinson’s disease.

Gene		MAF < 1%				MAF < 0.1%			
		Missense	Dmis	LoF	LoF + Dmis	Missense	Dmis	LoF	LoF + Dmis
*TMEM230*	WES cohort	9 (0.147)	6 (0.874)	–	6 (0.874)	6 (0.648)	4 (0.475)	–	4 (0.475)
	WGS cohort	10 (0.755)	6 (0.355)	–	6 (0.355)	7 (0.829)	4 (0.456)	–	4 (0.456)
*TMEM59*	WES cohort	**14 (0.035)**	12 (0.069)	1 (0.635)	13 (0.071)	**13 (0.007)**	**11 (0.016)**	1 (0.635)	**12 (0.030)**
	WGS cohort	12 (0.582)	11 (0.420)	1 (0.125)	12 (0.191)	11 (0.383)	10 (0.216)	1 (0.125)	11 (0.071)
*TMEM108*	WES cohort	27 (0.259)	17 (0.444)	–	17 (0.444)	24 (0.691)	17 (0.444)	–	17 (0.444)
	WGS cohort	27 (0.071)	12 (0.526)	1 (0.413)	13 (0.551)	**24 (0.014)**	12 (0.526)	1 (0.413)	13 (0.551)
*TMEM163*	WES cohort	11 (0.511)	10 (0.510)	–	10 (0.510)	11 (0.511)	10 (0.510)	–	10 (0.510)
	WGS cohort	8 (0.689)	8 (0.689)	–	8 (0.689)	7 (0.603)	7 (0.603)	–	7 (0.603)
*TMEM175*	WES cohort	51 (0.151)	29 (0.592)	7 (0.618)	36 (0.601)	42 (0.284)	25 (0.729)	6 (0.492)	31 (0.820)
	WGS cohort	49 (0.396)	25 (0.646)	4 (0.182)	29 (0.441)	40 (0.299)	21 (0.630)	3 (0.095)	24 (0.343)
*TMEM229B*	WES cohort	6 (0.403)	5 (0.442)	–	5 (0.442)	6 (0.403)	5 (0.442)	–	5 (0.442)
	WGS cohort	5 (0.764)	4 (0.830)	1 (0.582)	5 (0.885)	5 (0.764)	4 (0.830)	1 (0.582)	5 (0.885)

*Data were show as Number of Variants (SKAT-O P-value). MAF, minor allele frequency; Dmis, damaging missense variant (predicted by CADD ≥ 12.37); LoF, loss of function variant. Bold text indicates a statistically significant association with a p-value less than 0.05.*

**TABLE 2:** Parkinson’s disease (PD) patient specific variants of *TMEM230.*

Gene	Position (hg19)	Ref	Alt	NM number	AAChange	Consequence	gnomAD_ exome_ EAS^a^	gnomAD_ genome_ EAS^a^	ExAC_EAS^a^	CADD	WES cohort		WGS cohort	
											Case (*n* = 1917)	Control (*n* = 1652)	Case (*n* = 1962)	Control (*n* = 1279)
*TMEM230*	20:5081468	G	A	NM_001009923	c.521C > T:p.S174F	Missense	–	–	–	31	0	0	1	0
*TMEM230*	20:5081495	T	C	NM_001009923	c.494A > G:p.Y165C	Missense	0.0002	–	0.0001	21.1	0	0	2	0
*TMEM230*	20:5093650	C	G	NM_001009923	c.25G > C:p.V9L	Missense	–	–	–	22.7	0	0	1	0

*^a^Variants minor allele frequencies from gnomAD_genome_EAS, gnomAD_exome_EAS, and ExAC_EAS. AAChange, amino acid change.*

We identified 14 rare missense variants, including 12 Dmis variants and one LoF variant for *TMEM59* in the WES cohort with MAF < 1%. Gene-based burden analysis showed that the rare missense variants of *TMEM59* in the WES cohort were suggestively associated with PD (*P* = 0.035). When the MAF was limited to less than 0.1%, the association of 13 rare missense variants of *TMEM59* with PD became statistically significant (*P* = 0.007). At the same time, the 11 rare Dmis variants (*P* = 0.016) and the 12 Dmis plus LoF variants (*P* = 0.030) of *TMEM59* both became suggestively associated with PD in the WES cohort (Table 1; Figure 1B). Furthermore, 24 PD cases carried rare Dmis or LoF variants of *TMEM59*, so we listed their corresponding clinical manifestations in Supplementary Table 4. All those patients showed good responses to L-dopa treatment, and 10 of 24 cases showed dyskinesia. Besides, 13 and 12 rare non-synonymous variants were identified in the WGS cohort at the threshold of MAF < 1% and 0.1%, respectively. However, the association of these variants with PD was not statistically significant (Table 1; Figure 1B).

Twenty-seven rare missense variants were identified in both WES and WGS cohorts at MAF < 1%, and 24 rare missense variants were identified in both cohorts at MAF < 0.1% for *TMEM108*. 17 Dmis variants were found in the WES cohort with MAF < 1% or MAF < 0.1%, while 12 Dmis and one LoF variant were found in the WGS cohort with two MAF thresholds (Table 1; Figure 1C). Besides, gene-based burden analysis showed that the rare missense variants of *TMEM108* from the WGS cohort with MAF < 0.1% were suggestively associated with PD (*P* = 0.014).

*TMEM175* had the most missense variants at two different thresholds of MAF. Fifty-one in the WES cohort and 49 in the WGS cohort with MAF < 1%, and 42 in the WES cohort and 40 in the WGS cohort with MAF < 0.1%. However, the association of these variants with PD was not significantly different (Table 1; Figure 1E). Around or less than 10 rare missense variants were identified in both WES and WGS cohorts with two MAF thresholds for *TMEM163* and *TMEM229B* (Table 1; Figures 1D,F). Nevertheless, gene-based burden analysis did not find the association of these genes with PD, either.

Moreover, our study identified 20 PD families’ probands carrying rare predicted Dmis (MAF < 0.01 and CADD score over 12.37) or LoF variants. There are seven families with damaging variants predicted by two algorithms (CADD score over 12.37 and ReVe score over 0.7) discovered in FPD, and their corresponding pedigrees are shown in Supplementary Figure 2. Then, we conducted Sanger sequencing for the available family member of the corresponding family (AR-035); however, this variant did not co-segregate within the family.

## Discussion

To systematically investigate the genetic burden of six TMEM protein family genes, including *TMEM230*, *TMEM175*, *TMEM163*, *TMEM229B*, *TMEM108*, and *TMEM59*, in PD, we conducted a large case-control study in PD from the Chinese population, including FPD patients, sporadic early onset, and late-onset PD patients. Moreover, we mined the WES and WGS data, including rare and common variants covering coding, intron, and 2000 bp upstream and downstream of each gene, to explore these six genes. Then we found PD patient-specific rare variants for *TMEM230*, the significant association of rare missense variants of *TMEM59* with PD, and a suggestive association of rare missense variants of *TMEM108* with PD. However, no common variant of these genes was found to be significantly associated with PD.

We identified several PD-specific rare non-synonymous variants for *TMEM230*, but no significant association was found between this gene with PD. The p.Y165C variant was previously identified in a Taiwanese sporadic PD patient, while p.S174F and p.V9L variants have not previously been reported in PD patients or healthy controls (Quadri et al., 2017). According to the American College of Medical Genetics and Genomics (ACMG) guidelines, these variants were predicted to be uncertain significance due to a lack of supporting evidence. Hence, verifications of these variants in more studies and related functional investigations are warranted to confirm their pathogenicity in the future. *TMEM230* mutations were reported to cause autosomal dominant PD with the potential mechanism of impairing synaptic vesicle trafficking, disrupting mitochondria transport, and inducing apoptotic cell death (Deng et al., 2016; Wang et al., 2020, 2021a). Of note, TMEM230 protein is present in SNCA-positive Lewy bodies and Lewy neurites, indicating its potentially vital role in PD pathogenesis (Deng et al., 2016). There have been several subsequent verifications of the pathogenicity of the *TMEM230* gene in PD. However, many of them have not been verified (Yan et al., 2017; Wang et al., 2021b), which may be due to the relatively small contribution of the *TMEM230* gene in PD patients. In our recent large-cohort study, we identified one pathogenic variant (c.429delT/p.P144Qfs*2) of *TMEM230*, supporting the pathogenic role of *TMEM230* in PD (Zhao et al., 2020).

Then, we explored the role of common and rare variants in the other five TMEM family genes located within known PD risk loci. This study is the first to report that rare variants of *TMEM59* were significantly enriched in FPD and sEOPD patients, indicating *TMEM59* might play an essential role in PD. *TMEM59* has differentially expressed in substantia nigra pars compacta astrocytes in a PD mouse model (Khan et al., 2022). Besides, TMEM59 interacted with TREM2, which was also related to PD, and regulated microglia function (Liu et al., 2020). *TMEM59* was found to mediate PD-related pathways, such as autophagy and dopamine system dysfunction (Boada-Romero et al., 2013; Liu et al., 2017); moreover, overexpression of *TMEM59* in a *Drosophila* PD model ameliorated the phenotype of shortened lifespan, impaired locomotor activity, and loss of dopaminergic neurons by degrading a-synuclein (Zhang et al., 2018). Combining all the evidence, we supported that the rare variants of *TMEM59* might predictably increase the risk of PD. Since this is the first study to report the association of *TMEM59* with PD, genetic analysis studies for *TMEM59* in PD from larger cohorts and different populations are warranted to verify our findings.

We also found the suggestive association of *TMEM108* with PD. *TMEM108* was reported to be enriched in the dentate gyrus and involved in regulating spine development and glutamatergic activity in the granule neurons of the dentate gyrus (Jiao et al., 2017). Moreover, *TMEM108* was found to play a vital role in adult neurogenesis in the dentate gyrus (Yu et al., 2019). A large longitudinal study showed that *TMEM108* variant rs138073281 was associated with cognitive progression in PD (Zhang et al., 2021). However, no association of the *TMEM108* gene with PD was found in previous studies, and no functional study has been conducted to investigate the role of *TMEM108* in PD pathogenesis. Further research for *TMEM108* in PD should focus on these genetic analysis studies and functional investigations.

Our study identified several rare non-synonymous *TMEM175*, *TMEM163*, and *TMEM229B* variants, but no association of these genes with PD was found. TMEM175 is a component of a lysosomal K^+^ channel, and its deficiency results in impaired lysosomal and mitochondrial function (Jinn et al., 2017; Pergel et al., 2021). Variants of *TMEM175* have been identified to be associated with PD in a GWAS study, indicating its role in PD (Nalls et al., 2014). Further studies found that both rare and common variants of *TMEM175* were correlated to PD (Hopfner et al., 2020; Rudakou et al., 2021), and different common variants of *TMEM175* may have opposite influences on stress-induced damage and α-synuclein accumulation in neurons, which complicated the specific role of *TMEM175* in PD (Wie et al., 2021). It may explain why we could not find the association of *TMEM175* with PD at the gene level. TMEM163 is a zinc efflux transporter and belongs to the cation diffusion facilitator (CDF) protein family (Sanchez et al., 2019; Styrpejko and Cuajungco, 2021). The mutations of CDF family member, *SLC30A10*, were reported to cause parkinsonism (Quadri et al., 2012). *TMEM163* is highly expressed in the cortex and cerebellum, and its expression was positively associated with PD risk (Cuajungco et al., 2014; Kia et al., 2021). The loci around *TMEM163* was reported to associate with PD in GWAS studies (Nalls et al., 2014; Bandrés-Ciga et al., 2016), but our study did not verify the association between *TMEM163* with PD. Hence further studies with larger sample sizes and different ethnic populations are needed. TMEM229B is a novel protein with unclear biological function. We did not find the association of *TMEM229B* with PD, and the association of *TMEM229B* locus with PD was controversial in previous studies (Nalls et al., 2014; Chang et al., 2017). So, *TMEM229B* may not play a vital role in PD, and functional studies for TMEM229B and its mutants are warranted to illustrate its role in PD better.

In addition, several studies investigated the gene expression profile in the human postmortem substantia nigra of PD patients, but the expression level of the six genes investigated in our study was not significantly different between PD and controls (Kelly et al., 2019; Nido et al., 2020). We also searched the six genes in BrainEXP-NPD, a website showing expression profiling in human brains for six neuropsychiatric disorders, including PD.^3^ No expression level for *TMEM230* was found in any brain region on the website. No significant difference between PD and controls for other five genes regarding the expression level in different brain regions was found (Supplementary Figure 3; Supplementary Table 5).

Our study has several limitations. Firstly, the statistical power of the analysis in our study was limited, even though we had a large case-control cohort. Secondly, non-coding variants cannot be detected by WES, so these variants were not analyzed in our sEOPD and FPD patients. Finally, our findings for the association of TMEM family genes with PD were only from genetic analysis and have not been further verified by functional studies.

In conclusion, we performed a large case-control study to systematically investigate the role of PD-related TMEM protein family genes in PD using WES and WGS with gene-based and allele-based burden analysis. We found three PD patient-specific variants of *TMEM230*, which need further verification for pathogenicity. Moreover, we identified the significant association of *TMEM59* with FPD and sEOPD, and a suggestively significant association of *TMEM108* with sLOPD. However, we did not find any significant association of common variants of TMEM family genes with PD. Our study shed light on the gene burden of TMEM protein family genes in PD, and furthermore extensive genetic analysis studies and functional experiments for these genes are warranted.

## Data Availability Statement

According to national legislation/guidelines, specifically the Administrative Regulations of the China on Human Genetic Resources (http://www.gov.cn/zhengce/content/2019-06/10/content_5398829.htm, http://english.www.gov.cn/policies/latest_releases/2019/06/10/content_281476708945462.htm), no additional raw data is available at this time. Data of this project can be accessed after an approval application to the China National GeneBank (CNGB) (https://db.cngb.org/cnsa/). Please refer to https://db.cngb.org/, or email: CNGBdb@cngb.org for detailed application guidance. The accession code CNP0003134 should be included in the application.

## Ethics Statement

The studies involving human participants were reviewed and approved by Ethics Committee of Xiangya Hospital of Central South University in China. The patients/participants provided their written informed consent to participate in this study.

## Author Contributions

YZ: design, methodology, and writing – original draft. KZ: design, execution, and writing – original draft. HP: methodology and writing. YW: blood samples collection and DNA extraction. XZ and QX: patients enrollment. YX: blood samples collection. QS: clinical data collection. JT: DNA extraction and writing – review. XY and JG: patients enrollment and clinical data collection. JL: methodology and writing – review. BT: conception and patients enrollment. ZL: design, conception, writing – review and editing, and supervision. All authors contributed to the article and approved the submitted version.

## Conflict of Interest

The authors declare that the research was conducted in the absence of any commercial or financial relationships that could be construed as a potential conflict of interest.

## Publisher’s Note

All claims expressed in this article are solely those of the authors and do not necessarily represent those of their affiliated organizations, or those of the publisher, the editors and the reviewers. Any product that may be evaluated in this article, or claim that may be made by its manufacturer, is not guaranteed or endorsed by the publisher.

## Footnotes

1
http://pd-mdcnc.com/

2
http://broadinstitute.github.io/picard/

3
http://brainexpnpd.org:8088/BrainEXPNPD/index.html
