# Supplementary material for: Genetic Analysis of Six Transmembrane Protein Family Genes in Parkinson’s Disease in a Large Chinese Cohort
Source: Front Aging Neurosci. 2022 Jul 4;14:889057. doi: 10.3389/fnagi.2022.889057 (PMC9289399; doi:10.3389/fnagi.2022.889057)

Supplementary Figure 1. Sanger sequencing results on several random rare damaging variants

A. Sanger sequencing verification in samples from WES cohorts

AR-035 (II5) TMEM175:NM_001297424:exon2:c.16G>A:p.V6M

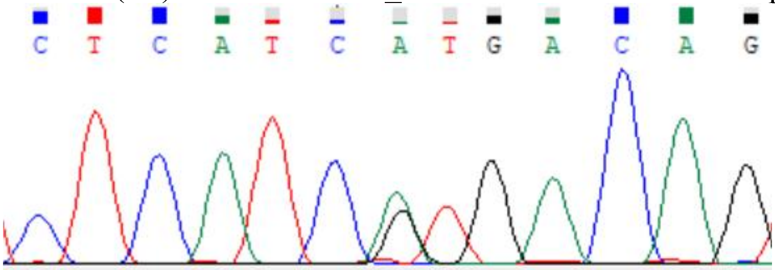

AR-035 (II7) TMEM175:NM_001297424:exon2:c.16G>A:p.V6M

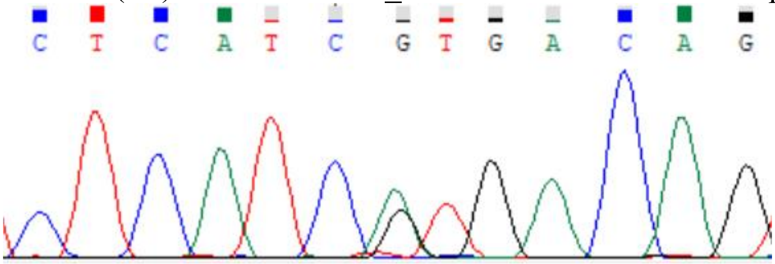

AR-035 (II8) TMEM175:NM_001297424:exon2:c.16G>A:p.V6M

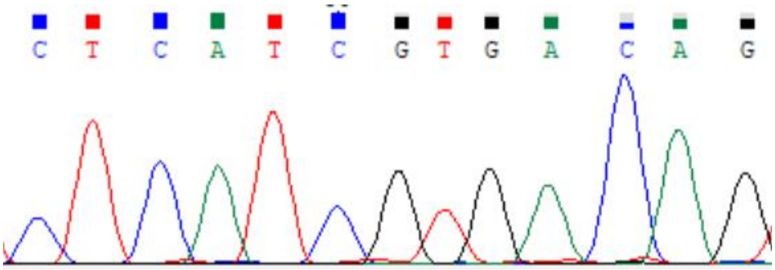

AR-035 (I1) TMEM175:NM_001297424:exon2:c.16G>A:p.V6M

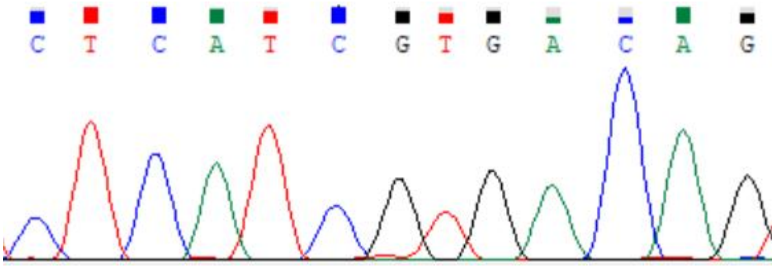

AR-035 (I2) TMEM175:NM_001297424:exon2:c.16G>A:p.V6M

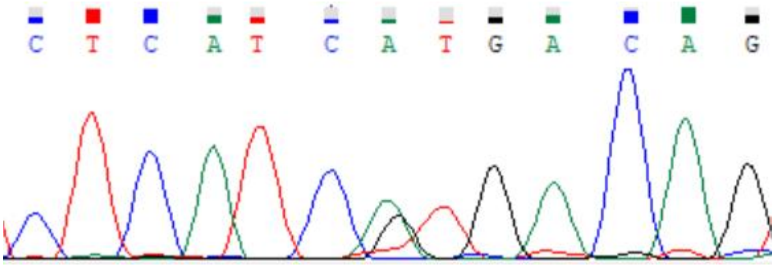

AR-035 (II2) TMEM175:NM_001297424:exon2:c.16G>A:p.V6M

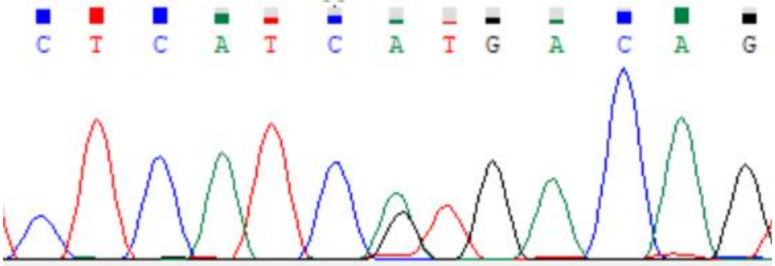

AR-035 (II4) TMEM175:NM_001297424:exon2:c.16G>A:p.V6M

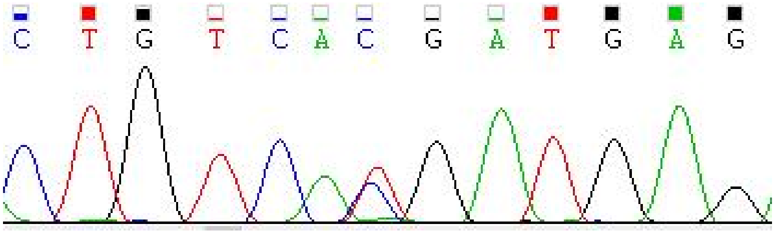

EOPD-0624 TMEM175:NM_001297424:exon9:c.659C>T:p.A220V

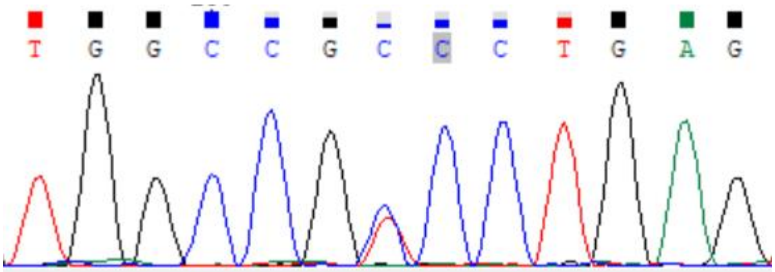

EOPD-0811 TMEM175:NM_001297424:exon5:c.137C>A:p.S46X

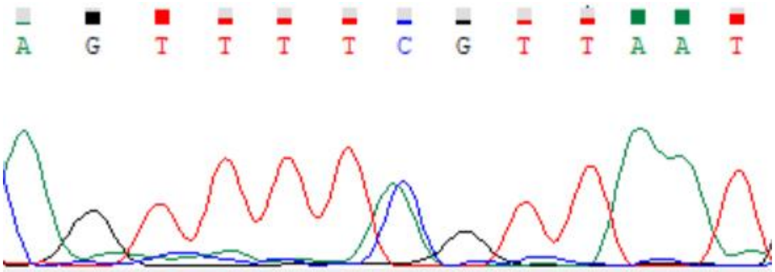

AD-076 TMEM175:NM_032326:exon2:c.72delG:p.D25Tfs*26

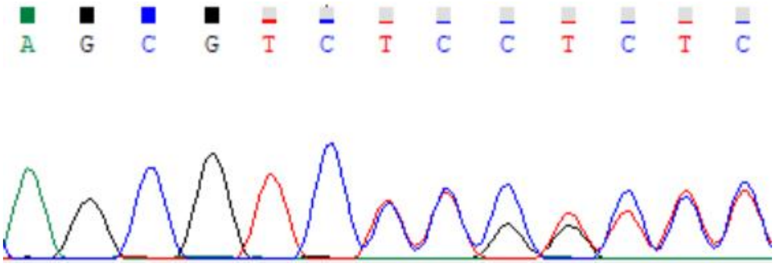

EOPD-0235 TMEM175:NM_001297424:exon2:c.16G>A:p.V6M

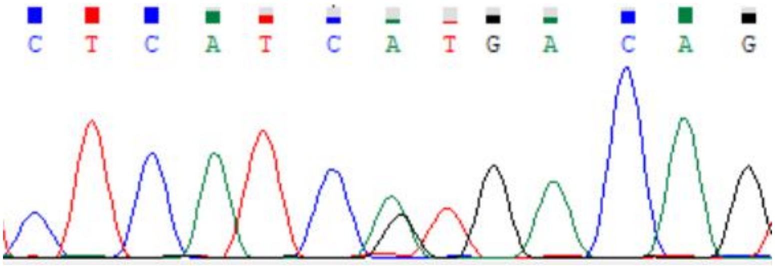

EOPD-0244 TMEM175:NM_001297424:exon8:c.574A>G:p.T192A

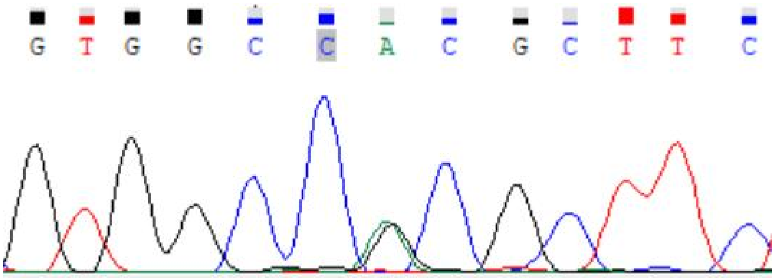

EOPD-0245 TMEM175:NM_001297424:exon8:c.533G>A:p.R178H

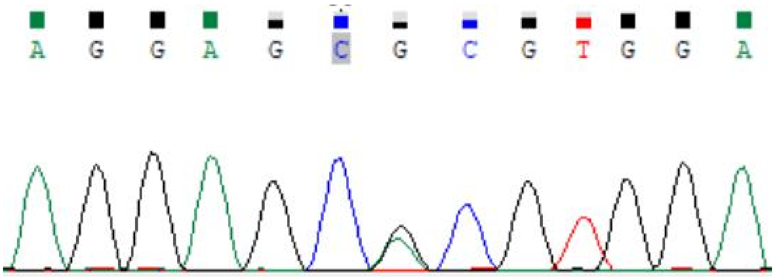

EOPD-0924 TMEM175:NM_032326:exon3:c.173C>T:p.T58M

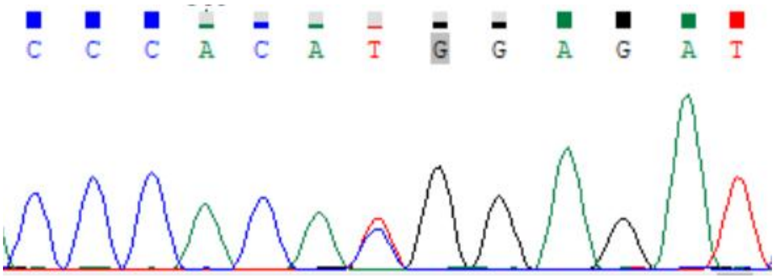

EOPD-1198 TMEM175:NM_001297424:exon9:c.782T>G:p.L261R

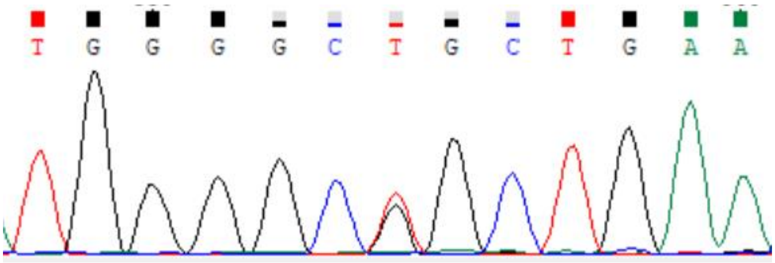

EOPD-0165 TMEM175:NM_001297424:exon8:c.526A>G:p.K176E

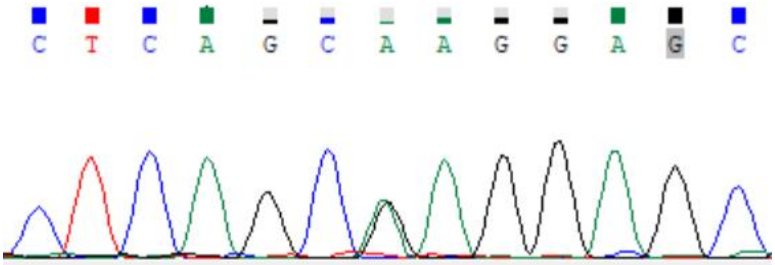

EOPD-1042 TMEM175:NM_032326:exon2:c.134C>T:p.S45F

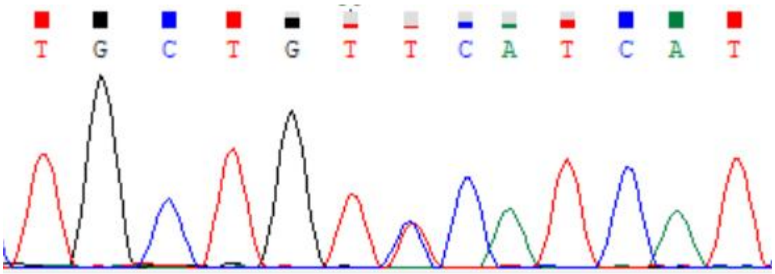

EOPD-1107 TMEM175:NM_001297424:exon9:c.1267T>C:p.X423Q

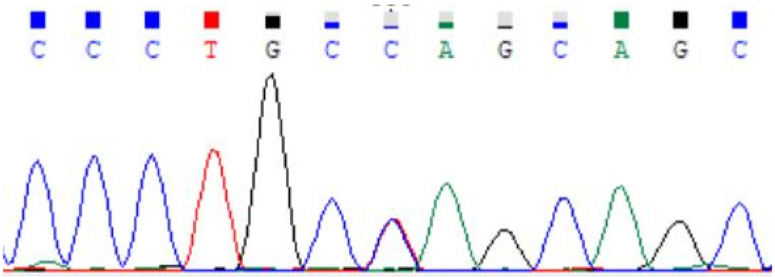

EOPD-1186 TMEM175:NM_001297424:exon9:c.1133T>C:p.L378P

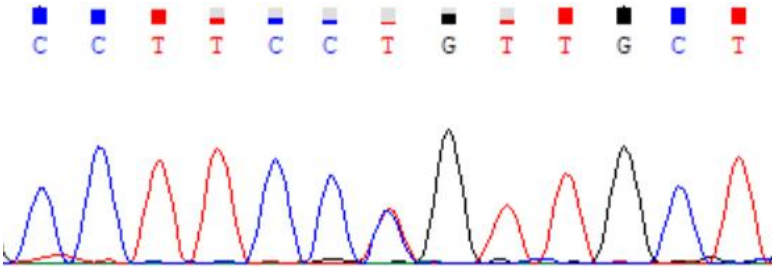

EOPD-1358 TMEM175:NM_001297424:exon9:c.1035_1036del:p.A347Qfs*119

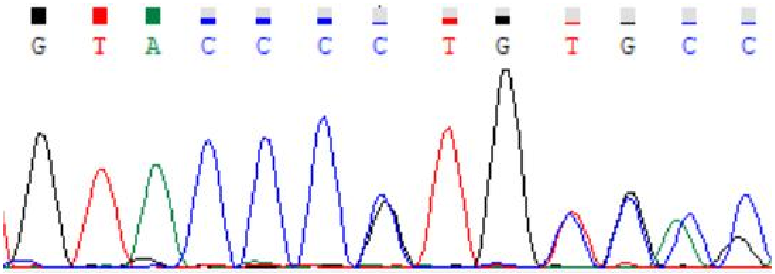

EOPD-1530 TMEM175:NM_001297424:exon8:c.526A>G:p.K176E

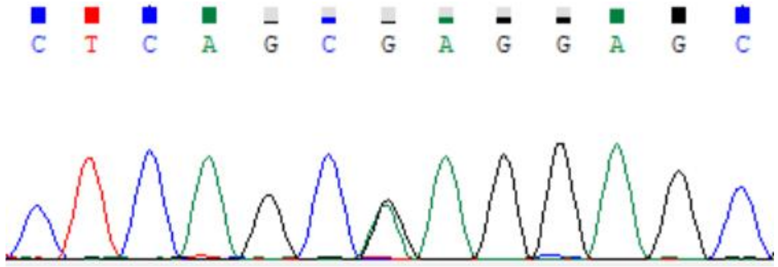

EOPD-0548 TMEM163:NM_030923:exon7:c.781G>A:p.G261S

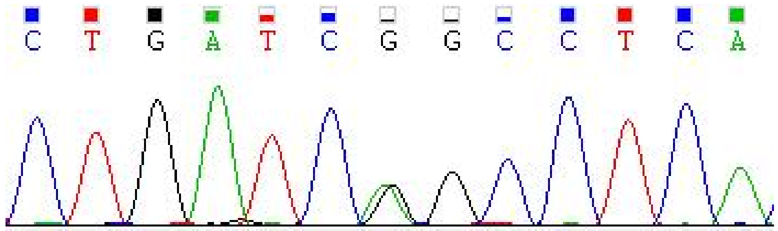

AD-101 TMEM108:NM_023943:exon4:c.56T>C:p.L19S

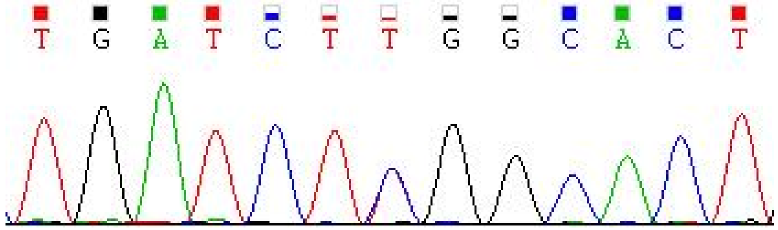

EOPD-0401 TMEM59:NM_004872:exon7:c.799C>T:p.Q267X

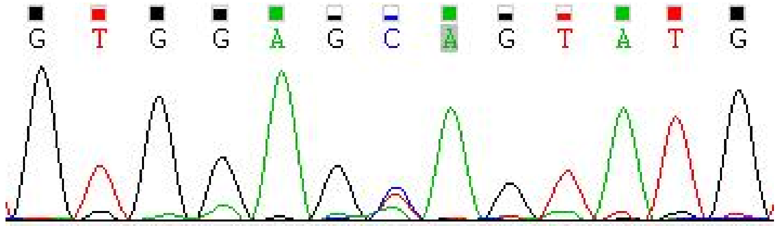

AD-189 TMEM59:NM_004872:exon7:c.799C>T:p.Q267X

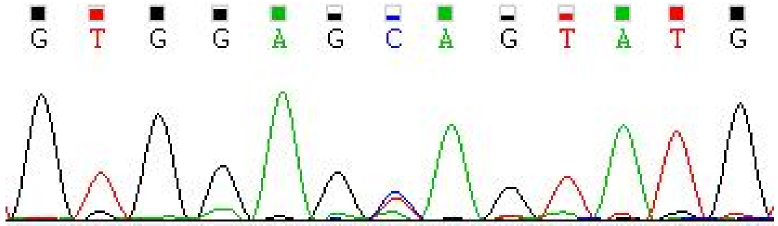

AD-210 TMEM229B:NM_001348549:exon4:c.34C>T:p.R12C

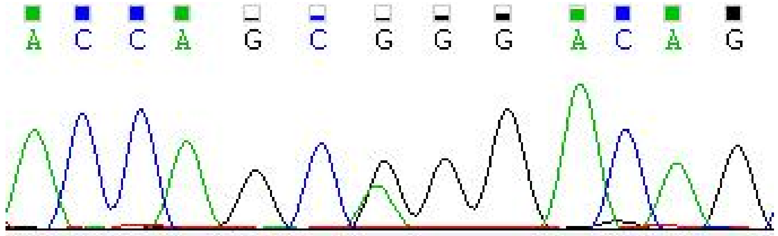

EOPD-1267 TMEM59:NM_004872:exon7:c.748G>T:p.V250L

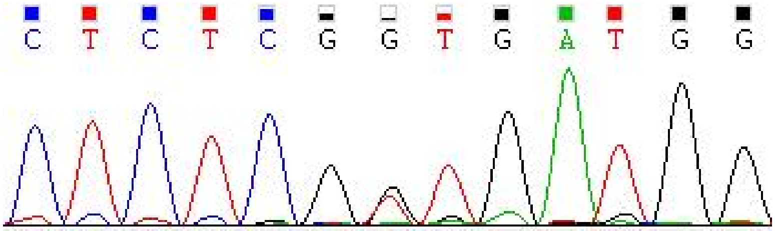

EOPD-1489 TMEM59:NM_004872:exon8:c.832G>A:p.G278S

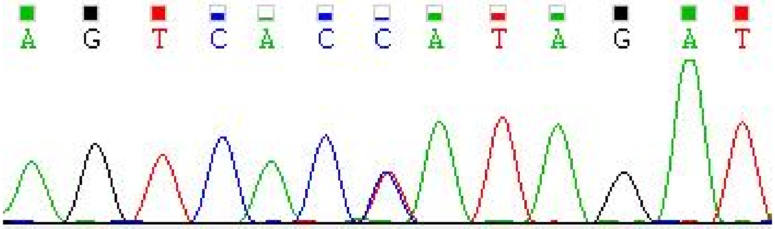

EOPD-1498 TMEM163:NM_030923:exon7:c.682G>A:p.V228M

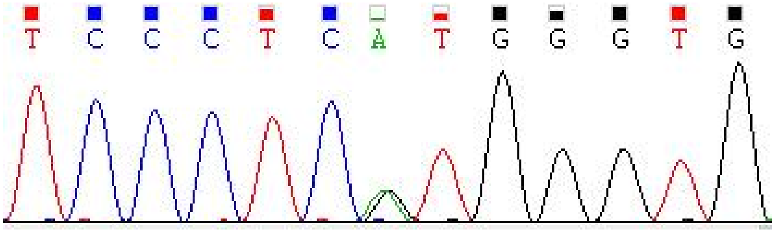

EOPD-1504 TMEM108:NM_023943:exon4:c.56T>C:p.L19S

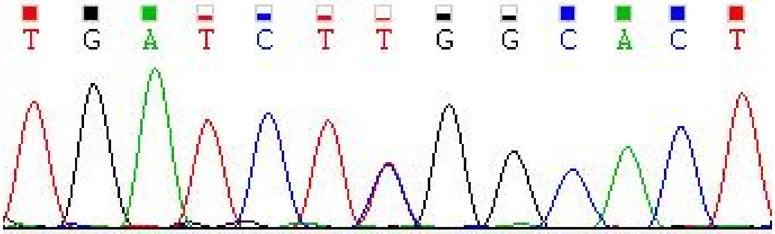

EOPD-0346 TMEM163:NM_030923:exon8:c.847C>G:p.R283G

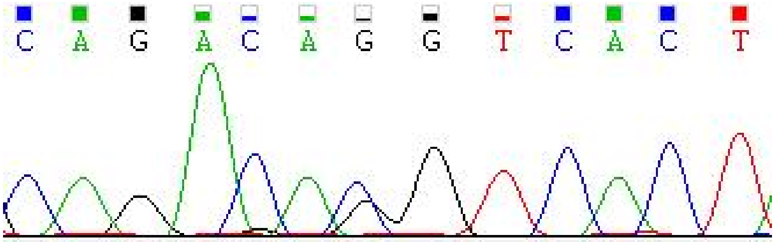

EOPD-1164 TMEM59:NM_004872:exon8:c.830A>G:p.Y277C

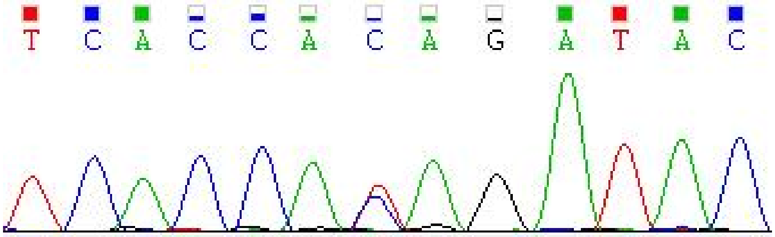

EOPD-0886 TMEM175:NM_001297426:exon10:c.283T>C:p.Y95H

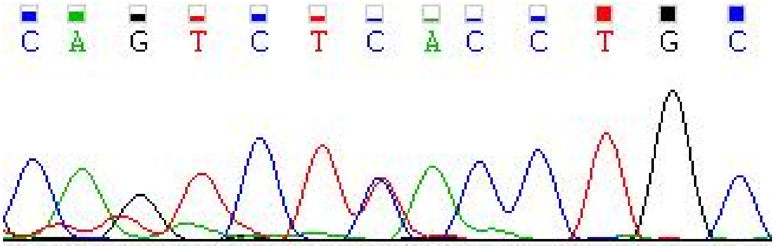

B. **Sanger sequencing verification in samples from WGS cohorts**

hn0317 TMEM175:NM_001297424:exon8:c.526A>G:p.K176E

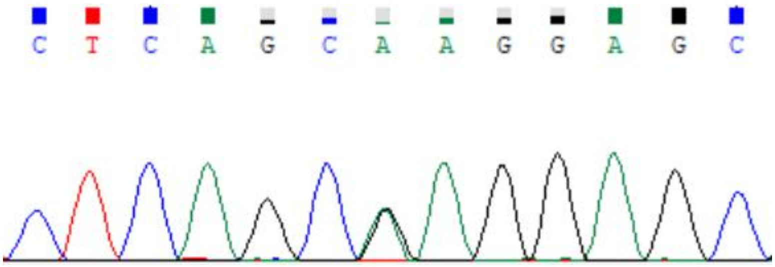

hn0696 TMEM175:NM_001297424:exon9:c.1267T>C:p.X423Q

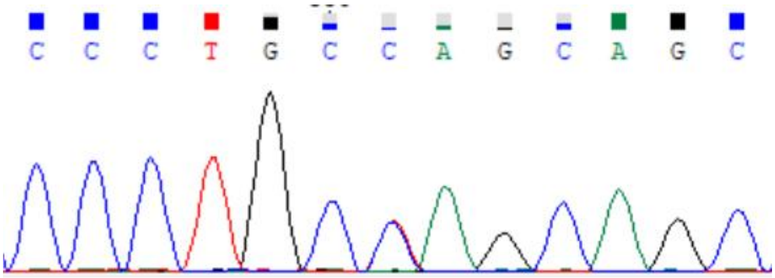

hn0719 TMEM175:NM_001297424:exon9:c.1073T>A:p.L358Q

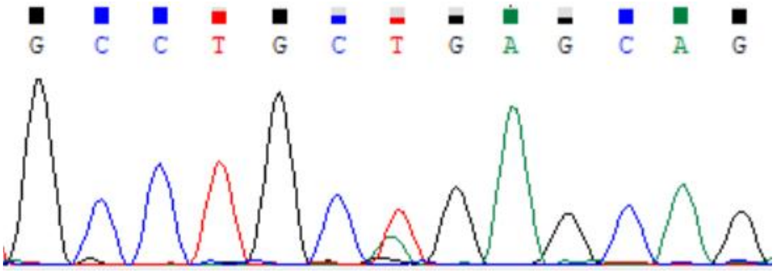

hn0794 TMEM175:NM_001297424:exon8:c.526A>G:p.K176E

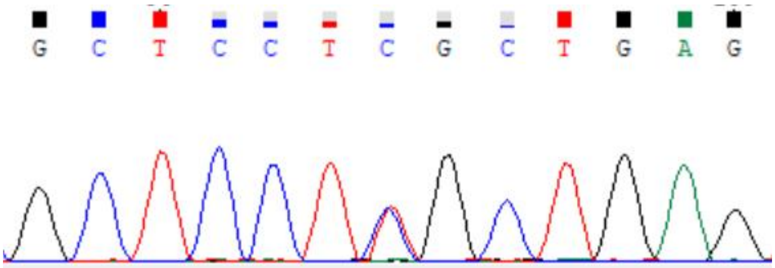

hn1248 TMEM175:NM_001297424:exon9:c.1133T>C:p.L378P

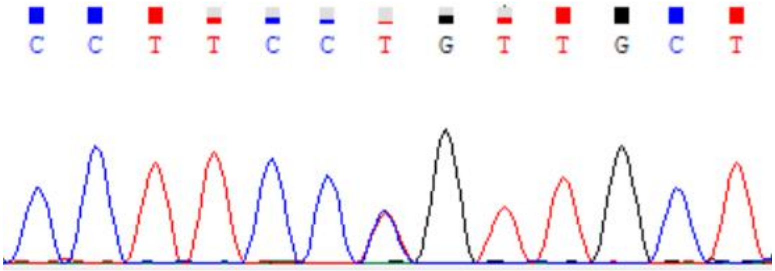

hn1314 TMEM175:NM_032326:exon2:c.72delG:p.D25Tfs*26

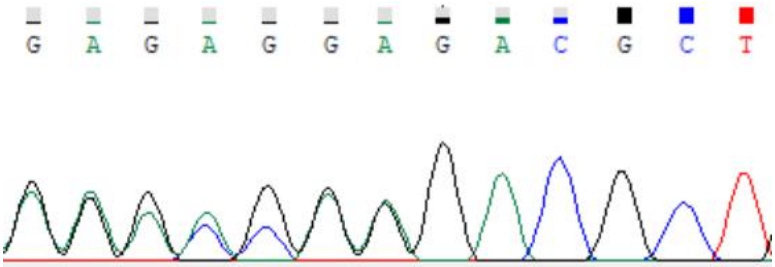

hn1386 TMEM175:NM_001297424:exon8:c.526A>G:p.K176E

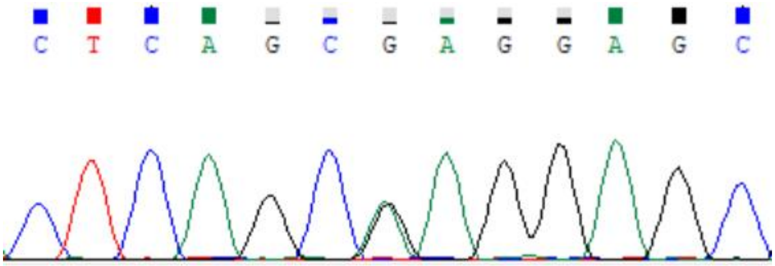

hn1573 TMEM175:NM_001297424:exon8:c.526A>G:p.K176E

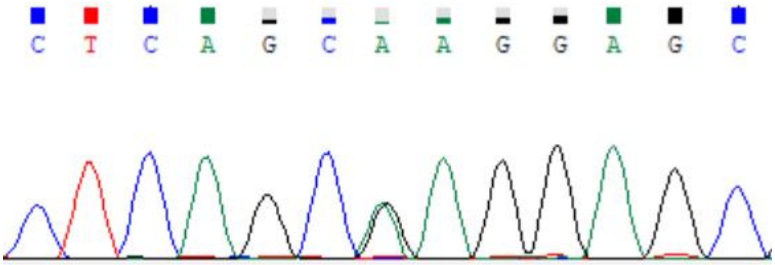

hn2862 TMEM175:NM_001297424:exon9:c.749T>A:p.L250Q

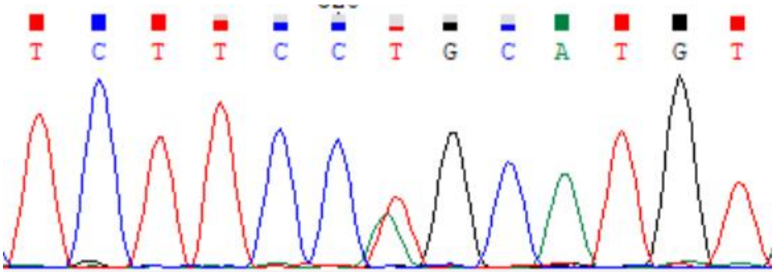

hn2897 TMEM175:NM_032326:exon4:c.218T>C:p.L73P

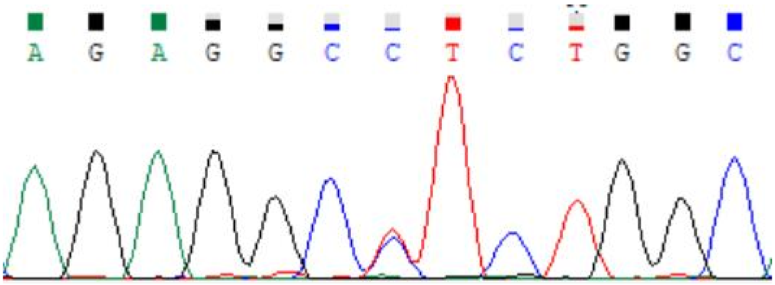

hn1835 TMEM175:NM_001297424:exon9:c.611C>T:p.P204L

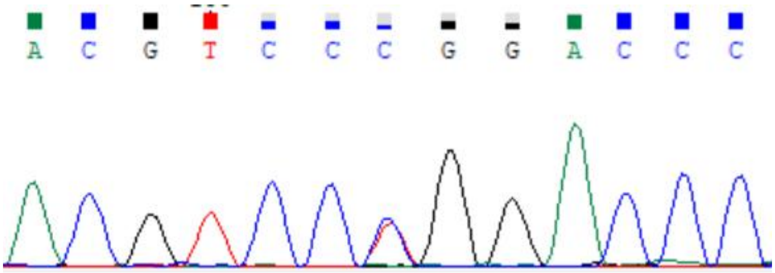

hn2959 TMEM175:NM_001297424:exon9:c.991G>A:p.G331S

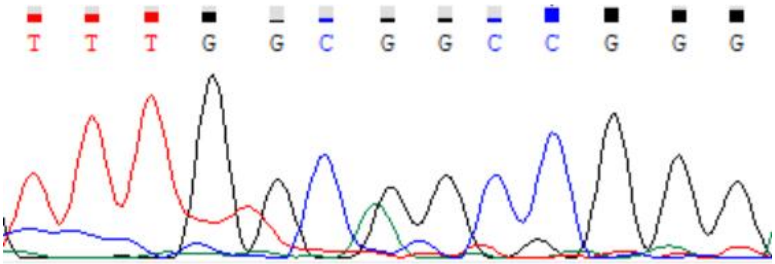

hn1909 TMEM175:NM_032326:exon2:c.72delG:p.D25Tfs*26

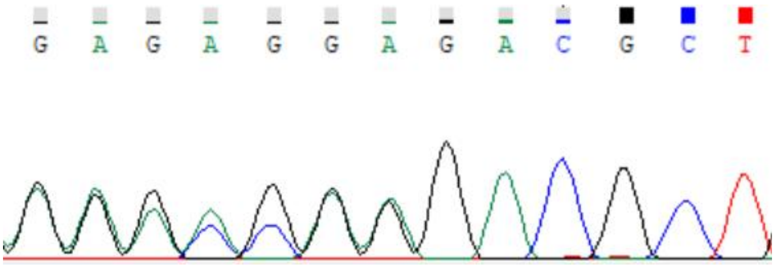

hn1913 TMEM175:NM_032326:exon2:c.72delG:p.D25Tfs*26

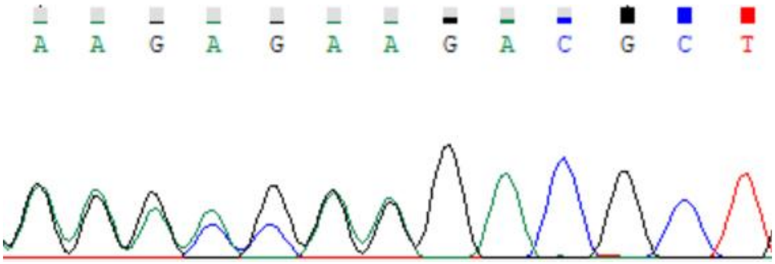

hn1921 TMEM175:NM_032326:exon2:c.84delG:p.I30Sfs*21

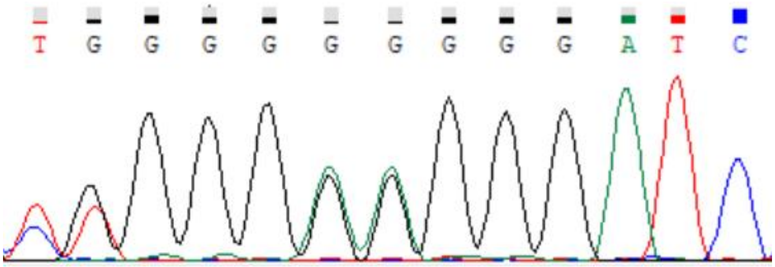

hn1989 TMEM175:NM_001297424:exon8:c.526A>G:p.K176E

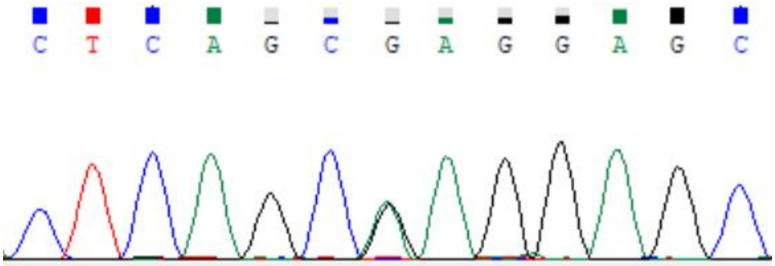

hn2800 TMEM59:NM_004872:exon7:c.799C>T:p.Q267X

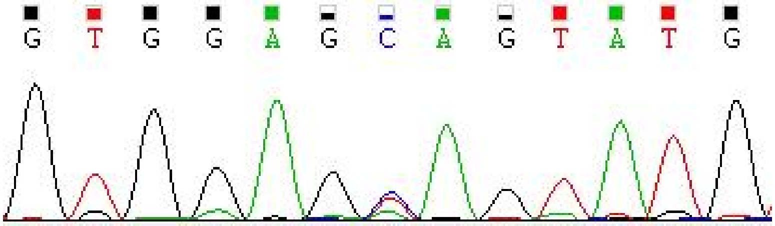

hn2865 TMEM59:NM_004872:exon7:c.799C>T:p.Q267X

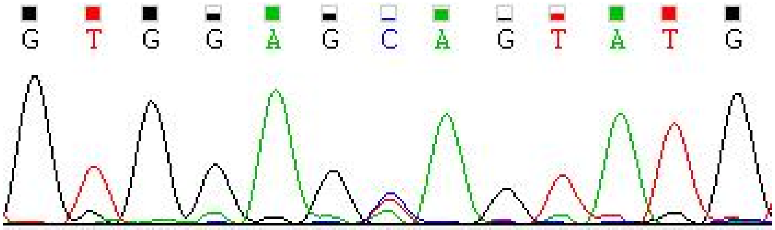

hn1720 TMEM59:NM_004872:exon7:c.799C>T:p.Q267X

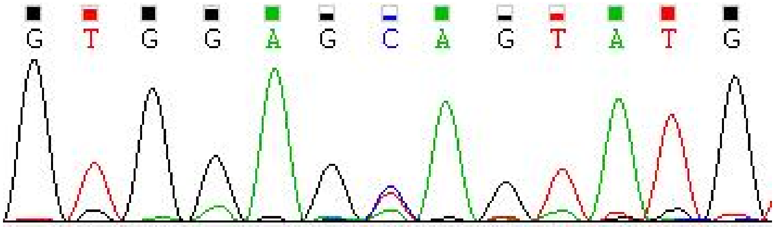

hn1748 TMEM108:NM_023943:exon4:c.56T>C:p.L19S

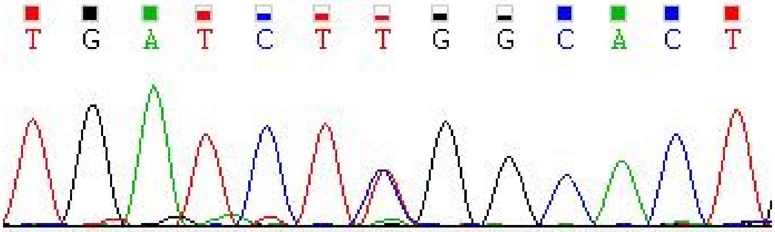

hn1761 TMEM108:NM_023943:exon4:c.56T>C:p.L19S

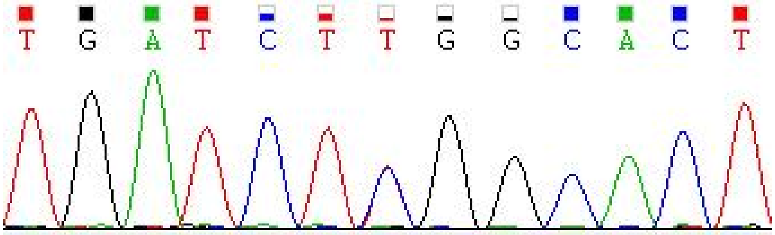

hn1964 TMEM163:NM_030923:exon7:c.719C>G:p.A240G

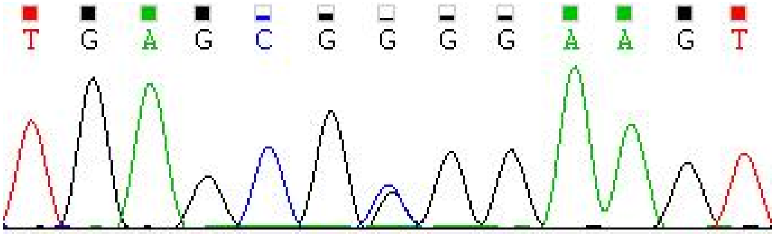

hn2659 TMEM59:NM_004872:exon4:c.439G>A:p.V147M

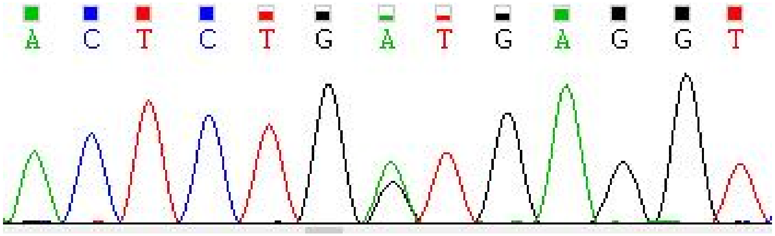

hn1872 TMEM163:NM_030923:exon5:c.484T>C:p.F162L

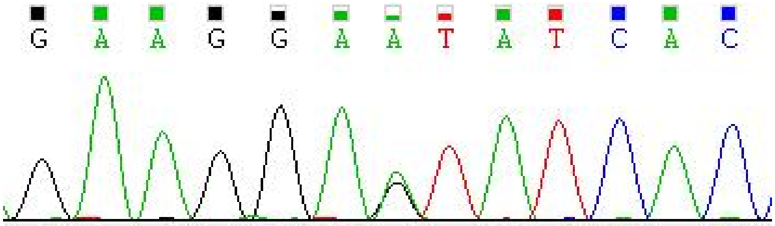

hn0843 TMEM175:NM_032326:exon4:c.262G>A:p.V88M

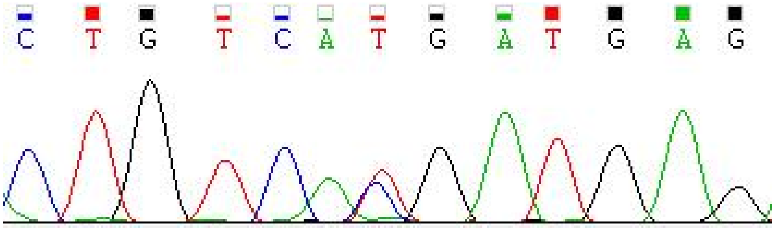

hn1461 TMEM59:NM_004872:exon8:c.836A>G:p.D279G

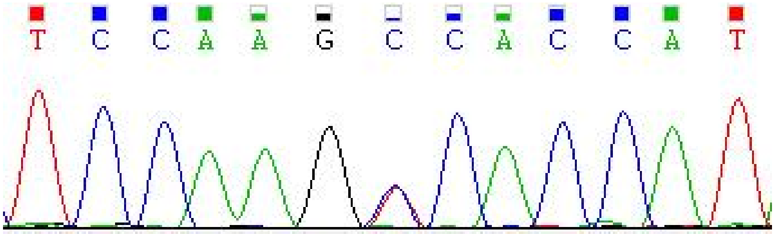

Supplement: Supplementary file 1 [file Data_Sheet_1.zip › Supplementary Figure 1.pdf]
