# Supplementary material for: Genetic Analysis of Six Transmembrane Protein Family Genes in Parkinson’s Disease in a Large Chinese Cohort
Source: Front Aging Neurosci. 2022 Jul 4;14:889057. doi: 10.3389/fnagi.2022.889057 (PMC9289399; doi:10.3389/fnagi.2022.889057)
Supplement: Supplementary file 1 [file Data_Sheet_1.zip › Supplementary Figure 3.pdf]

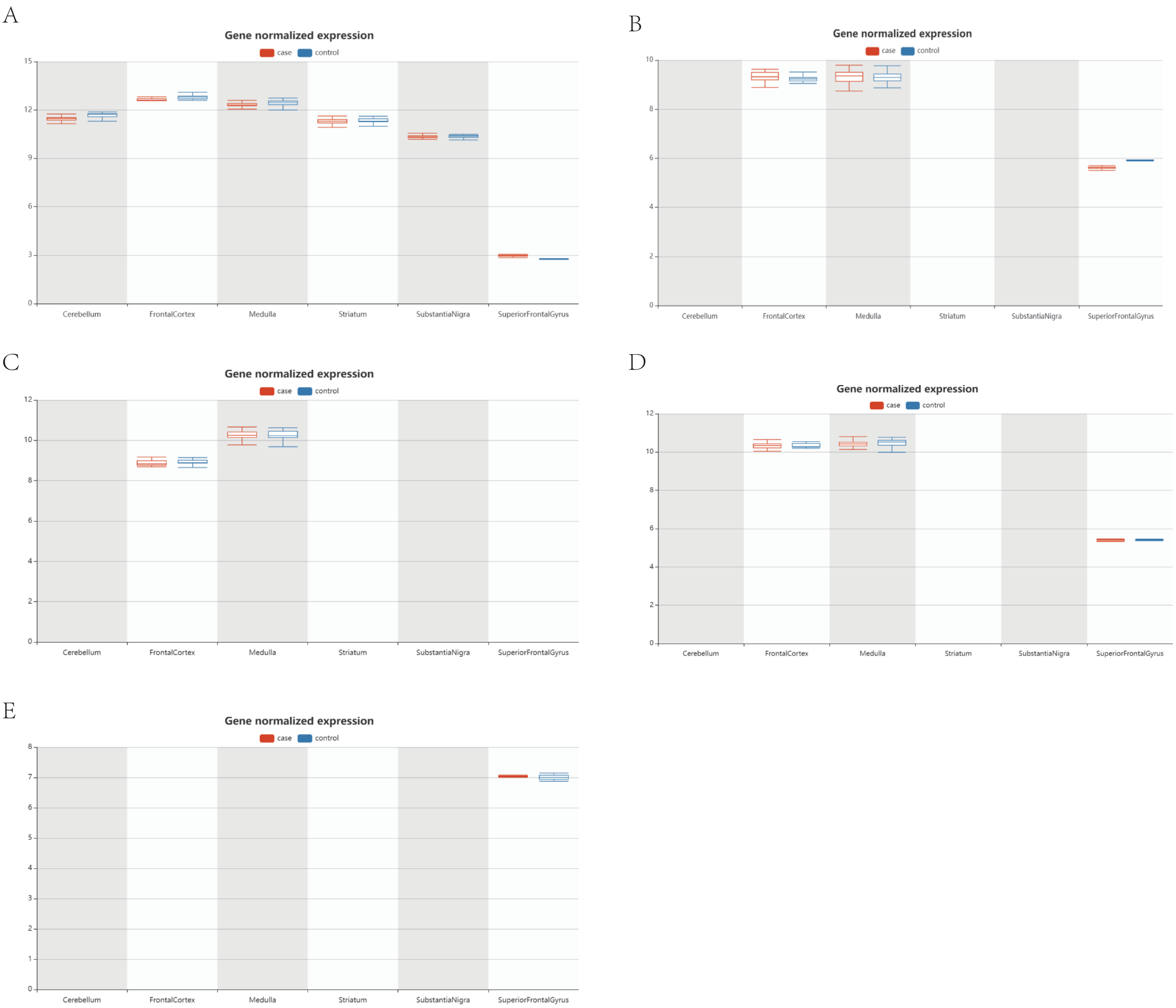

Supplementary Figure 3. Comparison of expression level for TMEM family genes between PD patients and controls in different brain regions. Shown are the box plots for comparisons of expression level of TMEM59 (A), TMEM108 (B), TMEM163 (C), TMEM175 (D), TMEM229B (E) in different brain regions between PD patients and controls. The figures were downloaded from BrainEXP-NPD, a website that shows the expression profiling in human brains for six neuropsychiatric disorders including PD (http://brainexpnpd.org:8088/BrainEXPNPD/index.html). No expression level for TMEM230 was found in any brain region on the website.
