# Supplementary material for: Genetic Analysis of Six Transmembrane Protein Family Genes in Parkinson’s Disease in a Large Chinese Cohort
Source: Front Aging Neurosci. 2022 Jul 4;14:889057. doi: 10.3389/fnagi.2022.889057 (PMC9289399; doi:10.3389/fnagi.2022.889057)
Supplement: Supplementary file 1 [file Data_Sheet_1.zip › Supplementary material.docx]

Supplementary Material

**Figure legend**

**Supplementary Figure 1.** Sanger sequencing results on several random rare damaging variants

**Supplementary Figure 2.** The family pedigrees of familial PD patients with damaging variants predicted by both two algorithms

**Supplementary Figure 3.** Comparison of expression level for TMEM family genes between PD patients and controls in different brain regions. Shown are the box plots for comparisons of expression level of *TMEM59* (**A**), *TMEM108* (**B**), *TMEM163* (**C**), *TMEM175* (**D**), *TMEM229B* (**E**) in different brain regions between PD patients and controls. The figures were downloaded from BrainEXP-NPD, a website that shows the expression profiling in human brains for six neuropsychiatric disorders including PD (http://brainexpnpd.org:8088/BrainEXPNPD/index.html). No expression level for TMEM230 was found in any brain region on the website.
