# Supplementary material for: Genetic Analysis of Six Transmembrane Protein Family Genes in Parkinson’s Disease in a Large Chinese Cohort
Source: Front Aging Neurosci. 2022 Jul 4;14:889057. doi: 10.3389/fnagi.2022.889057 (PMC9289399; doi:10.3389/fnagi.2022.889057)
Supplement: Supplementary file 1 [file Data_Sheet_1.zip › Supplementary Table 1.docx]

**Supplementary Table 1. Basic demographic characteristics of included subjects.**

| **Cohorts** | **WES cohort** | | | |  | **WGS cohort** | |
| --- | --- | --- | --- | --- | --- | --- | --- |
|  | AD probands  (n = 327) | AR probands  (n = 150) | sEOPD  (n = 1,440) | Control group 1  (n = 1,652) |  | sLOPD  (n = 1,962) | Control group 2  (n = 1,279) |
| Age | 56.74±11.66 | 60.7±11.3 | 50.25±7.14 | 62.03±12.59 |  | 66.76±7.08 | 62.32±7.11 |
| Age at onset | 51.78±10.8 | 54.98±11.98 | 44.17±5.79 | - |  | 61.88±6.93 | - |
| Sex (male/female) | 180/147 | 82/68 | 786/654 | 795/857 |  | 984/978 | 613/666 |
