# Supplementary material for: Genetic Analysis of Six Transmembrane Protein Family Genes in Parkinson’s Disease in a Large Chinese Cohort
Source: Front Aging Neurosci. 2022 Jul 4;14:889057. doi: 10.3389/fnagi.2022.889057 (PMC9289399; doi:10.3389/fnagi.2022.889057)
Supplement: Supplementary file 1 [file Data_Sheet_1.zip › Supplementary Table 2.docx]

**Supplementary Table 2. Summary of targeted genes in this study**

| **Gene** | **Location** | **OMIM** | **Note ^*^** |
| --- | --- | --- | --- |
| *TMEM230* | 20p13-p12.3 | 617019 | Disease-causing gene of PD [9] |
| *TMEM59* | 1p32.3 | 617084 | Functionally related to PD [20] |
| *TMEM108* | 3q22.1 | 617361 | Cognitive progression loci for PD [17] |
| *TMEM163* | 2q21.3 | 618978 | GWAS locus for PD [13] |
| *TMEM175* | 4p16.3 | 616660 | GWAS locus for PD [13] |
| *TMEM229B* | 14q24.1 | 619022 | GWAS locus for PD [13] |

^*^Denotes the potential association with neurodegenerative diseases
