# Supplementary material for: Genetic Analysis of Six Transmembrane Protein Family Genes in Parkinson’s Disease in a Large Chinese Cohort
Source: Front Aging Neurosci. 2022 Jul 4;14:889057. doi: 10.3389/fnagi.2022.889057 (PMC9289399; doi:10.3389/fnagi.2022.889057)
Supplement: Supplementary file 1 [file Data_Sheet_1.zip › Supplementary Table 3.docx]

**Supplementary Table 3. Rare damaging variants of TMEM family genes identified in our cohort**

| **Gene** | **Position (hg19)** | **Ref** | **Alt** | **NM number** | **AAChange** | **Consequence** | **gnomAD_exome_EAS ^a^** | **gnomAD_genome_EAS ^a^** | **ExAC_EAS ^a^** | **CADD** | **WES cohort** | | **WGS cohort** | |
| --- | --- | --- | --- | --- | --- | --- | --- | --- | --- | --- | --- | --- | --- | --- |
|  |  |  |  |  |  |  |  |  |  |  | **Case (n=1917)** | **Control (n=1652)** | **Case (n=1962)** | **Control (n=1279)** |
| *TMEM230* | 20:5081468 | G | A | NM_001009923 | c.521C>T:p.S174F | missense | - | - | - | 31 | 0 | 0 | 1 | 0 |
| *TMEM230* | 20:5081495 | T | C | NM_001009923 | c.494A>G:p.Y165C | missense | 0.0002 | - | 0.0001 | 21.1 | 0 | 0 | 2 | 0 |
| *TMEM230* | 20:5081564 | G | C | NM_001009923 | c.425C>G:p.A142G | missense | - | - | - | 25.4 | 0 | 0 | 1 | 1 |
| *TMEM230* | 20:5090063 | C | T | NM_001009923 | c.203G>A:p.R68H | missense | 0 | 0 | 0 | 24.4 | 0 | 1 | 0 | 0 |
| *TMEM230* | 20:5090066 | G | A | NM_001009923 | c.200C>T:p.S67F | missense | - | - | - | 29.8 | 0 | 2 | 0 | 0 |
| *TMEM230* | 20:5093607 | C | T | NM_001009923 | c.68G>A:p.R23Q;- | missense | 0.0021 | 0.0012 | 0.0091 | 26.6 | 5 | 3 | 2 | 4 |
| *TMEM230* | 20:5093629 | C | A | NM_001009923 | c.46G>T:p.G16W | missense | 0.00009815 | - | - | 23.3 | 2 | 1 | 0 | 0 |
| *TMEM230* | 20:5093647 | C | T | NM_001009923 | c.28G>A:p.G10S | missense | 0 | - | - | 17.89 | 0 | 1 | 0 | 0 |
| *TMEM230* | 20:5093650 | C | G | NM_001009923 | c.25G>C:p.V9L | missense | - | - | - | 22.7 | 0 | 0 | 1 | 0 |
| *TMEM230* | 20:5093667 | G | C | NM_001009923 | c.8C>G:p.P3R | missense | 0.0005 | 0.0018 | - | 18.93 | 1 | 0 | 1 | 1 |
| *TMEM59* | 1:54497869 | G | C | NM_001305043 | c.929C>G:p.A310G | missense | - | - | - | 26.3 | 1 | 0 | 0 | 0 |
| *TMEM59* | 1:54497906 | C | T | NM_001305043 | c.892G>A:p.V298M | missense | 0 | - | - | 21.4 | 0 | 1 | 1 | 0 |
| *TMEM59* | 1:54497959 | T | C | NM_001305043 | c.839A>G:p.D280G | missense | 0 | - | 0 | 28.6 | 0 | 0 | 1 | 0 |
| *TMEM59* | 1:54497963 | C | T | NM_001305043 | c.835G>A:p.G279S | missense | 0.00005845 | - | - | 33 | 1 | 0 | 0 | 0 |
| *TMEM59* | 1:54497965 | T | C | NM_001305043 | c.833A>G:p.Y278C | missense | 0.00005844 | 0 | 0.0001 | 27 | 1 | 0 | 0 | 0 |
| *TMEM59* | 1:54502300 | G | A | NM_001305043 | c.802C>T:p.Q268X | stopgain | 0.0001 | - | 0.0001 | 45 | 4 | 3 | 4 | 0 |
| *TMEM59* | 1:54502345 | C | T | NM_001305043 | c.757G>A:p.V253I | missense | 0.0013 | 0.0012 | 0.0012 | 34 | 7 | 6 | 5 | 4 |
| *TMEM59* | 1:54502345 | C | A | NM_001305043 | c.757G>T:p.V253L | missense | 0.00005851 | - | - | 34 | 0 | 1 | 1 | 0 |
| *TMEM59* | 1:54502351 | C | A | NM_001305043 | c.751G>T:p.V251L | missense | - | - | - | 33 | 1 | 0 | 0 | 0 |
| *TMEM59* | 1:54502372 | T | C | NM_001305043 | c.730A>G:p.T244A | missense | - | - | - | 22.7 | 0 | 0 | 0 | 1 |
| *TMEM59* | 1:54506486 | G | A | NM_001305043 | c.653C>T:p.A218V | missense | 0 | 0 | 0 | 17.29 | 1 | 0 | 0 | 0 |
| *TMEM59* | 1:54507466 | T | C | NM_001305043 | c.556A>G:p.I186V | missense | 0.0003 | - | 0.0001 | 12.47 | 1 | 0 | 0 | 0 |
| *TMEM59* | 1:54509150 | C | T | NM_001305043 | c.439G>A:p.V147M | missense | 0.00006734 | - | 0.0001 | 31 | 0 | 0 | 1 | 0 |
| *TMEM59* | 1:54511402 | T | G | NM_001305043 | c.353A>C:p.Q118P | missense | 0.00005798 | - | 0.0001 | 24.2 | 0 | 0 | 0 | 1 |
| *TMEM59* | 1:54512963 | C | T | NM_001305043 | c.272G>A:p.R91Q | missense | 0 | 0 | 0 | 22.5 | 1 | 0 | 0 | 0 |
| *TMEM59* | 1:54518673 | C | A | NM_001305043 | c.189G>T:p.K63N | missense | 0.0002 | - | 0.0001 | 26.4 | 0 | 0 | 1 | 0 |
| *TMEM59* | 1:54518675 | T | C | NM_001305043 | c.187A>G:p.K63E | missense | 0.00005825 | - | - | 21.3 | 0 | 0 | 1 | 0 |
| *TMEM59* | 1:54518725 | G | A | NM_001305043 | c.137C>T:p.A46V | missense | 0.0009 | 0 | 0.0008 | 21.9 | 5 | 0 | 5 | 2 |
| *TMEM59* | 1:54518764 | G | A | NM_001305043 | c.98C>T:p.A33V | missense | - | - | - | 14.75 | 1 | 0 | 0 | 0 |
| *TMEM59* | 1:54518816 | G | A | NM_001305043 | c.46C>T:p.L16F | missense | - | - | - | 22.9 | 0 | 0 | 1 | 0 |
| *TMEM163* | 2:135214307 | G | A | NM_030923 | c.850C>T:p.H284Y | missense | - | - | - | 27.4 | 0 | 1 | 1 | 0 |
| *TMEM163* | 2:135214310 | G | C | NM_030923 | c.847C>G:p.R283G | missense | - | - | - | 34 | 1 | 0 | 0 | 0 |
| *TMEM163* | 2:135214330 | A | G | NM_030923 | c.827T>C:p.V276A | missense | 0.00005801 | - | - | 24.7 | 1 | 1 | 0 | 0 |
| *TMEM163* | 2:135215631 | C | T | NM_030923 | c.781G>A:p.G261S | missense | 0 | - | 0 | 28.7 | 1 | 0 | 0 | 0 |
| *TMEM163* | 2:135215672 | G | A | NM_030923 | c.740C>T:p.S247L | missense | 0 | - | 0 | 22.2 | 1 | 0 | 0 | 0 |
| *TMEM163* | 2:135215690 | T | G | NM_030923 | c.722A>C:p.E241A | missense | - | - | - | 23.2 | 1 | 0 | 0 | 0 |
| *TMEM163* | 2:135215693 | G | C | NM_030923 | c.719C>G:p.A240G | missense | - | - | - | 22.1 | 0 | 0 | 1 | 0 |
| *TMEM163* | 2:135215694 | C | T | NM_030923 | c.718G>A:p.A240T | missense | 0 | 0 | 0 | 17.39 | 0 | 1 | 0 | 0 |
| *TMEM163* | 2:135215709 | A | T | NM_030923 | c.703T>A:p.S235T | missense | - | - | - | 21.6 | 0 | 0 | 1 | 0 |
| *TMEM163* | 2:135215730 | C | T | NM_030923 | c.682G>A:p.V228M | missense | 0 | - | - | 28.1 | 1 | 0 | 0 | 0 |
| *TMEM163* | 2:135223793 | C | T | NM_030923 | c.559G>A:p.D187N | missense | 0 | - | 0 | 24.2 | 1 | 0 | 0 | 0 |
| *TMEM163* | 2:135260543 | A | G | NM_030923 | c.484T>C:p.F162L | missense | - | - | - | 26.3 | 0 | 0 | 1 | 0 |
| *TMEM163* | 2:135308141 | A | G | NM_030923 | c.458T>C:p.I153T | missense | 0 | - | - | 20.9 | 0 | 0 | 0 | 1 |
| *TMEM163* | 2:135309654 | C | T | NM_030923 | c.331G>A:p.V111I | missense | 0 | - | 0 | 17.57 | 0 | 1 | 0 | 1 |
| *TMEM163* | 2:135476348 | C | T | NM_030923 | c.158G>A:p.R53Q | missense | 0 | 0.0019 | - | 24.3 | 0 | 0 | 1 | 1 |
| *TMEM163* | 2:135476408 | G | C | NM_030923 | c.98C>G:p.P33R | missense | - | - | - | 13.66 | 0 | 0 | 6 | 3 |
| *TMEM108* | 3:132764745 | G | A | NM_023943 | UTR5 | splicing | - | - | - | - | 0 | 0 | 1 | 0 |
| *TMEM108* | 3:133098611 | T | C | NM_023943 | c.56T>C:p.L19S | missense | 0.00005798 | 0.0006 | - | 24.4 | 2 | 0 | 3 | 0 |
| *TMEM108* | 3:133098644 | A | G | NM_023943 | c.89A>G:p.Q30R | missense | - | - | - | 15.75 | 1 | 0 | 0 | 0 |
| *TMEM108* | 3:133099025 | C | A | NM_023943 | c.470C>A:p.P157H | missense | 0 | 0 | 0 | 15.42 | 0 | 1 | 0 | 0 |
| *TMEM108* | 3:133099025 | C | T | NM_023943 | c.470C>T:p.P157L | missense | 0 | - | - | 16.22 | 2 | 0 | 0 | 1 |
| *TMEM108* | 3:133099031 | G | A | NM_023943 | c.476G>A:p.R159H | missense | 0.00005998 | - | 0 | 16.33 | 0 | 0 | 1 | 0 |
| *TMEM108* | 3:133099043 | G | T | NM_023943 | c.488G>T:p.R163L | missense | - | - | - | 22.8 | 0 | 0 | 1 | 0 |
| *TMEM108* | 3:133099096 | C | T | NM_023943 | c.541C>T:p.R181C | missense | 0 | - | 0 | 28 | 0 | 0 | 0 | 1 |
| *TMEM108* | 3:133099147 | C | G | NM_023943 | c.592C>G:p.R198G | missense | - | - | - | 22.4 | 1 | 0 | 0 | 0 |
| *TMEM108* | 3:133099187 | G | A | NM_023943 | c.632G>A:p.R211Q | missense | 0 | 0 | 0 | 25.3 | 0 | 1 | 0 | 0 |
| *TMEM108* | 3:133099410 | G | C | NM_023943 | c.855G>C:p.Q285H | missense | 0.0001 | - | 0.0001 | 18.64 | 0 | 1 | 0 | 0 |
| *TMEM108* | 3:133099523 | G | T | NM_023943 | c.968G>T:p.G323V | missense | - | - | - | 21.1 | 1 | 1 | 0 | 0 |
| *TMEM108* | 3:133099607 | G | T | NM_023943 | c.1052G>T:p.G351V | missense | 0.0001 | - | - | 16.52 | 1 | 0 | 1 | 0 |
| *TMEM108* | 3:133099630 | C | T | NM_023943 | c.1075C>T:p.P359S | missense | 0.00005798 | - | - | 13.1 | 1 | 1 | 0 | 1 |
| *TMEM108* | 3:133099712 | C | G | NM_023943 | c.1157C>G:p.P386R | missense | 0.0002 | - | - | 17.66 | 3 | 0 | 0 | 0 |
| *TMEM108* | 3:133099909 | G | C | NM_023943 | c.1354G>C:p.E452Q | missense | - | - | - | 24.2 | 0 | 1 | 0 | 1 |
| *TMEM108* | 3:133099922 | C | T | NM_023943 | c.1367C>T:p.P456L | missense | 0 | - | 0 | 33 | 0 | 0 | 1 | 0 |
| *TMEM108* | 3:133109068 | G | A | NM_023943 | c.1495G>A:p.A499T | missense | 0.0001 | 0 | 0.0001 | 23.9 | 2 | 4 | 1 | 2 |
| *TMEM108* | 3:133109075 | C | T | NM_023943 | c.1502C>T:p.P501L | missense | 0 | - | 0 | 31 | 0 | 1 | 0 | 0 |
| *TMEM108* | 3:133114726 | C | T | NM_023943 | c.1624C>T:p.R542C | missense | 0 | - | 0.0001 | 28.6 | 1 | 0 | 1 | 0 |
| *TMEM108* | 3:133114730 | C | A | NM_023943 | c.1628C>A:p.S543Y | missense | - | - | - | 23.8 | 0 | 1 | 0 | 0 |
| *TMEM108* | 3:133114739 | A | G | NM_023943 | c.1637A>G:p.N546S | missense | 0 | - | 0 | 22.8 | 0 | 1 | 0 | 0 |
| *TMEM108* | 3:133114810 | G | A | NM_023943 | c.1708G>A:p.D570N | missense | 0.00006179 | 0 | 0 | 32 | 0 | 0 | 2 | 0 |
| *TMEM175* | 4:941561 | G | T | NM_032326 | c.34G>T:p.D12Y | missense | - | - | - | 19.43 | 0 | 0 | 1 | 1 |
| *TMEM175* | 4:941599 | G | - | NM_032326 | c.72delG:p.D25Tfs*26 | frameshift deletion | 0.0004 | - | 0.0002 | - | 1 | 2 | 5 | 0 |
| *TMEM175* | 4:941611 | G | - | NM_032326 | c.84delG:p.I30Sfs*21 | frameshift deletion | - | - | - | - | 0 | 0 | 1 | 0 |
| *TMEM175* | 4:941661 | C | T | NM_032326 | c.134C>T:p.S45F | missense | - | - | - | 32 | 1 | 0 | 0 | 0 |
| *TMEM175* | 4:941675 | G | A | NM_032326 | c.148G>A:p.V50I | missense | 0 | - | - | 24.7 | 0 | 1 | 0 | 0 |
| *TMEM175* | 4:941682 | T | C | NM_032326 | c.153+2T>C | splicing | - | - | - | 24.9 | 0 | 0 | 1 | 0 |
| *TMEM175* | 4:941923 | C | T | NM_032326 | c.173C>T:p.T58M | missense | 0 | 0 | 0 | 27.5 | 1 | 0 | 0 | 0 |
| *TMEM175* | 4:944234 | T | C | NM_032326 | c.218T>C:p.L73P | missense | - | - | - | 24.1 | 0 | 0 | 1 | 0 |
| *TMEM175* | 4:944246 | G | A | NM_032326 | c.230G>A:p.R77Q | missense | 0 | 0 | 0 | 24.4 | 0 | 1 | 1 | 0 |
| *TMEM175* | 4:944254 | G | A | NM_032326 | c.238G>A:p.V80I | missense | 0.00005798 | 0 | 0 | 15.85 | 1 | 0 | 0 | 0 |
| *TMEM175* | 4:944275 | A | C | NM_032326 | c.259A>C:p.I87L | missense | 0.0002 | - | 0.0002 | 13.82 | 6 | 3 | 3 | 4 |
| *TMEM175* | 4:944278 | G | A | NM_032326 | c.262G>A:p.V88M | missense | 0.0011 | 0.0012 | 0.0012 | 26 | 2 | 0 | 0 | 1 |
| *TMEM175* | 4:945042 | A | C | NM_032326 | c.338A>C:p.N113T | missense | 0 | 0 | 0 | 24.8 | 1 | 0 | 0 | 0 |
| *TMEM175* | 4:946159 | C | A | NM_032326 | c.383C>A:p.S128X | stopgain | - | - | - | 36 | 1 | 0 | 0 | 0 |
| *TMEM175* | 4:946202 | G | C | NM_032326 | c.426G>C:p.L142F | missense | 0.0005 | 0.0006 | 0.0005 | 23.7 | 2 | 0 | 0 | 1 |
| *TMEM175* | 4:946206 | TG | - | NM_032326 | c.430_431del:p.V147Dfs*103 | frameshift deletion | 0.0005 | 0.0006 | 0.0006 | - | 0 | 1 | 0 | 0 |
| *TMEM175* | 4:946222 | delete ^b^ | - | NM_032326 | c.446_462del:p.A149Gfs*96 | frameshift deletion | 0 | 0.0006 | 0 | - | 2 | 0 | 0 | 0 |
| *TMEM175* | 4:946233 | G | A | NM_032326 | c.457G>A:p.V153M | missense | 0.00005805 | 0 | 0 | 24.3 | 0 | 0 | 1 | 1 |
| *TMEM175* | 4:947024 | C | T | NM_032326 | c.509C>T:p.P170L | missense | 0 | 0 | 0 | 20.8 | 0 | 1 | 0 | 0 |
| *TMEM175* | 4:949196 | T | C | NM_032326 | c.631T>C:p.Y211H | missense | - | - | - | 24.2 | 1 | 0 | 0 | 0 |
| *TMEM175* | 4:949230 | A | G | NM_032326 | c.665A>G:p.Y222C | missense | 0.0002 | 0.0012 | 0 | 18.43 | 4 | 1 | 3 | 2 |
| *TMEM175* | 4:949582 | C | T | NM_032326 | c.746C>T:p.S249L | missense | 0.00005806 | 0 | 0.0001 | 21.4 | 0 | 1 | 0 | 0 |
| *TMEM175* | 4:949608 | A | G | NM_032326 | c.772A>G:p.K258E | missense | 0.0003 | - | 0.0005 | 25.1 | 4 | 2 | 5 | 2 |
| *TMEM175* | 4:949615 | G | A | NM_032326 | c.779G>A:p.R260H | missense | 0 | - | 0.0001 | 28.4 | 1 | 1 | 0 | 0 |
| *TMEM175* | 4:949635 | G | A | NM_032326 | c.799G>A:p.G267R | missense | 0 | - | 0 | 27.5 | 0 | 0 | 1 | 0 |
| *TMEM175* | 4:949656 | A | G | NM_032326 | c.820A>G:p.T274A | missense | - | - | - | 23.1 | 1 | 0 | 0 | 0 |
| *TMEM175* | 4:951626 | C | T | NM_032326 | c.857C>T:p.P286L | missense | 0 | - | 0 | 23.9 | 0 | 1 | 1 | 0 |
| *TMEM175* | 4:951632 | C | T | NM_032326 | c.863C>T:p.P288L | missense | 0.0007 | 0.0012 | 0.0012 | 24.9 | 0 | 2 | 0 | 2 |
| *TMEM175* | 4:951673 | G | A | NM_032326 | c.904G>A:p.A302T | missense | 0 | - | 0 | 23.8 | 0 | 0 | 1 | 0 |
| *TMEM175* | 4:951674 | C | T | NM_032326 | c.905C>T:p.A302V | missense | 0.00005816 | - | - | 25.8 | 1 | 0 | 0 | 0 |
| *TMEM175* | 4:951751 | CACT | - | NM_032326 | c.982_985del:p.L330Sfs*12 | frameshift deletion | - | - | - | - | 0 | 1 | 0 | 0 |
| *TMEM175* | 4:951764 | T | A | NM_032326 | c.995T>A:p.L332Q | missense | - | - | - | 23.7 | 0 | 0 | 1 | 0 |
| *TMEM175* | 4:951797 | T | G | NM_032326 | c.1028T>G:p.L343R | missense | 0.00005816 | - | - | 25.2 | 1 | 0 | 1 | 0 |
| *TMEM175* | 4:951847 | C | G | NM_032326 | c.1078C>G:p.Q360E | missense | - | - | - | 23.8 | 0 | 1 | 0 | 0 |
| *TMEM175* | 4:951895 | G | C | NM_032326 | c.1126G>C:p.V376L | missense | 0.00005851 | - | - | 18.6 | 0 | 0 | 1 | 0 |
| *TMEM175* | 4:951899 | G | A | NM_032326 | c.1130G>A:p.R377H | missense | 0.0002 | - | 0.0001 | 25.2 | 1 | 1 | 0 | 0 |
| *TMEM175* | 4:951959 | C | T | NM_032326 | c.1190C>T:p.A397V | missense | 0.0003 | 0.0006 | 0.0005 | 26 | 0 | 2 | 0 | 2 |
| *TMEM175* | 4:951965 | T | A | NM_032326 | c.1196T>A:p.L399Q | missense | - | - | - | 23.1 | 0 | 0 | 1 | 0 |
| *TMEM175* | 4:951980 | C | T | NM_032326 | c.1211C>T:p.T404M | missense | 0.0001 | 0 | 0.0001 | 22.7 | 0 | 1 | 0 | 0 |
| *TMEM175* | 4:952004 | G | A | NM_032326 | c.1235G>A:p.G412D | missense | 0 | - | 0 | 23.6 | 0 | 0 | 1 | 1 |
| *TMEM175* | 4:952006 | G | A | NM_032326 | c.1237G>A:p.G413S | missense | 0 | - | 0 | 23.4 | 0 | 0 | 1 | 0 |
| *TMEM175* | 4:952009 | C | T | NM_032326 | c.1240C>T:p.R414W | missense | 0.0005 | 0.0018 | 0.0006 | 24.7 | 3 | 3 | 1 | 0 |
| *TMEM175* | 4:952039 | G | A | NM_032326 | c.1270G>A:p.A424T | missense | 0.00005817 | - | 0.0001 | 19.1 | 0 | 1 | 0 | 0 |
| *TMEM175* | 4:952050 | CT | - | NM_032326 | c.1281_1282del:p.A429Qfs*119 | frameshift deletion | 0.0001 | 0 | 0.0004 | - | 1 | 0 | 0 | 0 |
| *TMEM175* | 4:952088 | T | A | NM_032326 | c.1319T>A:p.L440Q | missense | - | - | - | 23 | 0 | 0 | 0 | 2 |
| *TMEM175* | 4:952148 | T | C | NM_032326 | c.1379T>C:p.L460P | missense | - | - | - | 24.5 | 1 | 0 | 1 | 0 |
| *TMEM175* | 4:952157 | G | A | NM_032326 | c.1388G>A:p.R463H | missense | 0.00005842 | 0.0006 | 0 | 27.6 | 0 | 0 | 1 | 0 |
| *TMEM175* | 4:952210 | C | T | NM_032326 | c.1441C>T:p.R481W | missense | 0.0004 | 0 | 0.0003 | 23.2 | 1 | 0 | 0 | 0 |
| *TMEM175* | 4:952268 | T | A | NM_032326 | c.1499T>A:p.L500H | missense | - | - | - | 24.4 | 1 | 1 | 1 | 1 |
| *TMEM175* | 4:952282 | T | C | NM_032326 | c.1513T>C:p.X505Q | stoploss | 0.0004 | - | 0.0015 | 11.05 | 2 | 1 | 2 | 1 |
| *TMEM229B* | 14:67940171 | A | G | NM_001348543 | c.470T>C:p.L157P | missense | 0.0002 | - | 0.0001 | 19.16 | 3 | 0 | 1 | 1 |
| *TMEM229B* | 14:67940231 | C | T | NM_001348543 | c.410G>A:p.R137H | missense | - | - | - | 27.2 | 0 | 0 | 1 | 0 |
| *TMEM229B* | 14:67940390 | G | A | NM_001348543 | c.251C>T:p.T84I | missense | - | - | - | 13.49 | 0 | 0 | 0 | 1 |
| *TMEM229B* | 14:67940414 | C | A | NM_001348543 | c.227G>T:p.R76L | missense | - | - | - | 34 | 0 | 1 | 0 | 0 |
| *TMEM229B* | 14:67940433 | G | A | NM_001348543 | c.208C>T:p.R70C | missense | 0.00006227 | 0.0006 | - | 24.9 | 0 | 1 | 1 | 0 |
| *TMEM229B* | 14:67940553 | C | A | NM_001348543 | c.88G>T:p.A30S | missense | 0 | - | 0 | 23 | 0 | 1 | 0 | 0 |
| *TMEM229B* | 14:67940607 | G | A | NM_001348543 | c.34C>T:p.R12C | missense | 0 | - | - | 34 | 1 | 0 | 0 | 0 |
| *TMEM229B* | 14:67955226 | G | A | NM_001348543 | c.-193C>T | splicing | - | - | - | - | 0 | 0 | 1 | 0 |

1. Variants minor allele frequencies from gnomAD_genome_EAS, gnomAD_exome_EAS and ExAC_EAS.
2. The sequence of “delete” is CCATTGGGGTCGTGCAGGTAGGGGGCCTGGGGGGCCTGCACTGTGTGTGTGTGTGTGTGTGTGTGTGTGTGTGTGTGTGTGTGTGATCA
