# Supplementary material for: Genetic Analysis of Six Transmembrane Protein Family Genes in Parkinson’s Disease in a Large Chinese Cohort
Source: Front Aging Neurosci. 2022 Jul 4;14:889057. doi: 10.3389/fnagi.2022.889057 (PMC9289399; doi:10.3389/fnagi.2022.889057)
Supplement: Supplementary file 1 [file Data_Sheet_1.zip › Supplementary Table 4.docx]

**Supplementary Table 4. Clinical manifestations of PD carriers with *TMEM* gene rare damaging variants**

| Sample ID | AR-109 | EOPD-1489 | EOPD-1164 | AD-187 | AD-189 | EOPD-0401 | EOPD-0829 | AR-091 | EOPD-0027 | EOPD-0266 | EOPD-0675 | EOPD-1087 | EOPD-1417 | EOPD-1467 | EOPD-1267 | EOPD-1370 | EOPD-0102 | EOPD-0680 | AD-032 | AD-188 | EOPD-0468 | EOPD-0876 | EOPD-1204 | EOPD-0258 |  |
| --- | --- | --- | --- | --- | --- | --- | --- | --- | --- | --- | --- | --- | --- | --- | --- | --- | --- | --- | --- | --- | --- | --- | --- | --- | --- |
| Variant | c.929C>G | c.835G>A | c.833A>G | c.802C>T | c.802C>T | c.802C>T | c.802C>T | c.757G>A | c.757G>A | c.757G>A | c.757G>A | c.757G>A | c.757G>A | c.757G>A | c.751G>T | c.653C>T | c.556A>G | c.272G>A | c.137C>T | c.137C>T | c.137C>T | c.137C>T | c.137C>T | c.98C>T |  |
| Sex | M | F | M | M | M | F | M | F | F | F | F | M | M | M | M | M | F | F | M | F | F | F | F | M |  |
| AAO (years) | 65 | 49 | 43 | 56 | 57 | 43 | 50 | 61 | 47 | 48 | 36 | 36 | 50 | 48 | 48 | 50 | 46 | 48 | 49 | 70 | 49 | 37 | 43 | 45 |  |
| AAE (years) | 73 | 50 | 55 | 59 | 65 | 53 | 55 | 65 | 57 | 52 | 50 | 43 | 65 | 53 | 56 | 52 | 56 | 56 | 52 | 77 | 60 | 43 | 69 | 48 |  |
| Duration (years) | 8 | 1 | 12 | 3 | 8 | 10 | 5 | 4 | 10 | 4 | 14 | 7 | 15 | 5 | 8 | 2 | 10 | 8 | 3 | 7 | 11 | 6 | 26 | 3 |  |
| Hoehn-Yahr stage | 2.5 | 1.5 | 4.0 | 2.5 | 2.0 | 1.0 | 3.0 | 1.5 | 1.0 | 3.0 | 3.0 | 1.0 | 3.0 | 2.5 | 3.0 | 1.5 | 2.5 | 2.5 | 2.5 | 2.0 | 5.0 | 2.0 | 4.0 | 1.0 |  |
| Bradykinesia | + | + | + | + | + | + | + | + | + | + | + | + | + | + | + | + | + | + | + | + | + | + | + | + |  |
| Resting tremor | - | + | + | + | + | - | - | + | - | - | - | + | + | + | + | + | - | + | + | + | + | + | + | + |  |
| Rigidity | + | + | + | + | + | + | + | + | + | + | + | + | + | + | + | + | + | + | + | + | + | + | + | + |  |
| Postural instability | + | - | + | + | - | - | + | - | - | + | + | - | + | + | + | - | + | + | + | - | + | - | + | - |  |
| UPDRS part II | 14 | 5 | 21 | 10 | 13 | 7 | 19 | 8 | 10 | 10 | 15 | 7 | 11 | 9 | 26 | 10 | 8 | 24 | 10 | 10 | 32 | 9 | 18 | 10 |  |
| UPDRS part III | 25 | 14 | 64 | 32 | 34 | 7 | 36 | 19 | 11 | 23 | 41 | 13 | 23 | 20 | 60 | 14 | 23 | 55 | 31 | 29 | 74 | 26 | 26 | 17 |  |
| Motor subtype | PIGD | PIGD | PIGD | TD | PIGD | PIGD | PIGD | TD | PIGD | PIGD | PIGD | Intermittent | Intermittent | Intermittent | PIGD | PIGD | PIGD | TD | PIGD | PIGD | Intermittent | Intermittent | PIGD | Intermittent |  |
| Response to levodopa | Good | Good | Good | Good | Good | Good | Good | Good | Good | Good | Good | Good | Good | Good | Good | Good | Good | Good | Good | Good | Good | Good | Good | Good |  |
| Dyskinesia | - | + | + | - | + | + | - | - | - | - | + | - | + | + | + | - | + | - | - | - | + | - | - | - |  |
| Freezing gait | + | + | + | - | - | - | - | - | - | - | + | - | + | + | + | - | - | - | - | - | - | - | - | - |  |
| Hyposmia | + | + | + | NA | + | + | NA | NA | - | + | - | - | + | - | + | + | - | NA | NA | - | + | NA | - | - |  |
| Depression | - | - | + | - | + | + | - | - | + | + | + | - | - | + | + | - | - | NA | NA | + | + | NA | - | - |  |
| Constipation | + | - | + | - | - | - | + | NA | - | - | - | - | + | - | - | - | + | + | NA | + | + | + | + | - |  |
| Cognitive decline | - | - | - | - | - | - | NA | NA | - | - | NA | - | - | - | - | - | - | - | - | NA | NA | - | - | NA | NA |
| MMSE | 27 | 25 | 30 | 28 | 25 | 27 | NA | NA | 28 | 27 | NA | 25 | 27 | 27 | 25 | 30 | 28 | 25 | 27 | NA | NA | 28 | 27 | NA |  |
| pRBD | + | - | - | + | - | + | NA | NA | NA | - | + | - | + | - | - | - | - | NA | NA | + | + | NA | - | - |  |

AAO = Age at onset; AAE = Age at evaluation; UPDRS = Unified Parkinson’s Disease Rating Scale; MMSE = Mini-mental state examination; pRBD = probable Rapid eyes movement sleep behavior disorder; Disease motor subtype was classified as tremor-dominant (TD) phenotype when the ratio of tremor score and postural instability and gait difficulty (PIGD) score was no less than 1.5, while patients with a ratio of no more than 1.0 were defined to PIGD phenotype and rest of patients belonged to the indeterminate phenotype.
