# Supplementary material for: Genetic Analysis of Six Transmembrane Protein Family Genes in Parkinson’s Disease in a Large Chinese Cohort
Source: Front Aging Neurosci. 2022 Jul 4;14:889057. doi: 10.3389/fnagi.2022.889057 (PMC9289399; doi:10.3389/fnagi.2022.889057)
Supplement: Supplementary file 1 [file Data_Sheet_1.zip › Supplementary Table 5.docx]

**Supplementary Table 5. Expression level for TMEM family genes between PD patients and controls in different brain regions**

| **Brain Region** | **PD** | **Control** | **FDR** | |
| --- | --- | --- | --- | --- |
|  | **mean±sd** | **mean±sd** | **Mega** | **Meta** |
| ***TMEM59*** | | | | |
| Cerebellum | 11.458±0.160 | 11.667±0.199 | 0.048 | 0.106 |
| Frontal Cortex | 12.643±0.088 | 12.803±0.141 | 0.08 | |
| Medulla | 12.308±0.189 | 12.396±0.233 | 0.626 | |
| Striatum | 11.290±0.180 | 11.336±0.148 | 0.499 | 0.252 |
| Substantia Nigra | 10.350±0.108 | 10.361±0.092 | 0.799 | 0.954 |
| Superior Frontal Gyrus | 2.957±0.088 | 2.762±0.025 | 0.391 | |
| ***TMEM108*** | | | | |
| Frontal Cortex | 9.324±0.221 | 9.284±0.204 | 0.858 | |
| Medulla | 9.337±0.286 | 9.298±0.257 | 0.898 | |
| Superior Frontal Gyrus | 5.608±0.080 | 5.915±0.026 | 0.214 | |
| ***TMEM163*** | | | | |
| Frontal Cortex | 8.875±0.151 | 8.920±0.162 | 0.772 | |
| Medulla | 10.266±0.223 | 10.252±0.259 | 0.961 | |
| ***TMEM175*** | | | | |
| Frontal Cortex | 10.331±0.197 | 10.343±0.123 | 0.952 | |
| Medulla | 10.434±0.179 | 10.471±0.211 | 0.852 | |
| Superior Frontal Gyrus | 5.394±0.060 | 5.412±0.045 | 0.955 | |
| ***TMEM229B*** | | | | |
| Superior Frontal Gyrus | 7.037±0.032 | 7.008±0.137 | 0.937 | |

The above data was extracted from the BrainEXP-NPD, a website that shows the expression profiling in human brains for six neuropsychiatric disorders including PD (<http://brainexpnpd.org:8088/BrainEXPNPD/index.html>). No expression level for *TMEM230* was found in any brain region on the website. FDR shows the adjusted *P*-value after multiple testing correction.
